# Metatranscriptomics Reveals the RNA Virome of Ixodes Persulcatus in the China–North Korea Border, 2017

**DOI:** 10.3390/v16010062

**Published:** 2023-12-29

**Authors:** Ruichen Wang, Shenghui Liu, Hongliang Sun, Chongxiao Xu, Yanhan Wen, Xiwen Wu, Weijia Zhang, Kai Nie, Fan Li, Shihong Fu, Qikai Yin, Ying He, Songtao Xu, Guodong Liang, Liquan Deng, Qiang Wei, Huanyu Wang

**Affiliations:** 1 National Key Laboratory of Intelligent Tracking and Forecasting for Infectious Diseases, National Institute for Viral Disease Control and Prevention, Chinese Center for Disease Control and Prevention, Beijing 102206, China; wangrc96@163.com (R.W.); 17862970927@163.com (S.L.); xuchongxiao2021@163.com (C.X.); ryanwen94@163.com (Y.W.); zwj_0308@126.com (W.Z.); niekai@ivdc.chinacdc.cn (K.N.); lifan@ivdc.chinacdc.cn (F.L.); fush@ivdc.chinacdc.cn (S.F.); yinqk@ivdc.chinacdc.cn (Q.Y.); heying@ivdc.chinacdc.cn (Y.H.); xust@ivdc.chinacdc.cn (S.X.); lianggd@ivdc.chinacdc.cn (G.L.); 2 Changchun Institute of Biological Products Co., Ltd., Changchun 130012, China; sunhl1995@163.com (H.S.); wuxiwen@sinopharm.com (X.W.); 3 School of Public Health, Jilin University, Changchun 130021, China; 4 National Pathogen Resource Center, Chinese Center for Disease Control and Prevention, Beijing 102206, China

**Keywords:** China–North Korea border, *Ixodes persulcatus*, metatranscriptomics, tick-borne virus

## Abstract

In recent years, numerous viruses have been identified from ticks, and some have been linked to clinical cases of emerging tick-borne diseases. Chinese northeast frontier is tick infested. However, there is a notable lack of systematic monitoring efforts to assess the viral composition in the area, leaving the ecological landscape of viruses carried by ticks not clear enough. Between April and June 2017, 7101 ticks were collected to perform virus surveillance on the China–North Korea border, specifically in Tonghua, Baishan, and Yanbian. A total of 2127 *Ixodes persulcatus* were identified. Further investigation revealed the diversity of tick-borne viruses by transcriptome sequencing of *Ixodes persulcatus*. All ticks tested negative for tick-borne encephalitis virus. Transcriptome sequencing expanded 121 genomic sequence data of 12 different virus species from *Ixodes persulcatus*. Notably, a new segmented flavivirus, named Baishan Forest Tick Virus, were identified, closely related to Alongshan virus and Harz mountain virus. Therefore, this new virus may pose a potential threat to humans. Furthermore, the study revealed the existence of seven emerging tick-borne viruses dating back to 2017. These previously identified viruses included Mudanjiang phlebovirus, Onega tick phlebovirus, Sara tick phlebovirus, Yichun mivirus, and three unnamed viruses (one belonging to the *Peribunyaviridae* family and the other two belonging to the *Phenuiviridae* family). The existence of these emerging tick-borne viruses in tick samples collected in 2017 suggests that their history may extend further than previously recognized. This study provides invaluable insights into the virome of Ixodes persulcatus in the China–North Korea border region, enhancing our ongoing efforts to manage the risks associated with tick-borne viruses.

## 1. Introduction

Ticks, blood-sucking arthropods, have the ability to parasitize a wide range of animals, such as humans, livestock, and poultry [1]. Notably, ticks play a significant role as carriers and transmitters of viruses, potentially causing diseases in humans and other mammals through their blood-feeding activities. *Ixodes persulcatus* is a common species of tick and is one of the primary vectors for tick-borne encephalitis virus (TBEV) [2]. TBEV can lead to tick-borne encephalitis, a condition that poses a threat to the human central nervous system. Globally, there are around 10,000–12,000 reported cases of tick-borne encephalitis each year [3].

Furthermore, *I. persulcatus* has been identified as a carrier of viruses from at least seven different virus families, including *Peribunyaviridae*, *Nairoviridae*, *Orthomyxoviridae*, *Phenuiviridae*, *Reoviridae*, *Flaviviridae*, and *Phenuiviridae* [4,5]. Some of these viruses, such as TBEV and Powassan virus (POWV), are closely associated with human diseases. Although the transmission efficiency of tick-borne viruses is not as high as that of respiratory viruses such as SARS-CoV-2 or influenza virus, the diseases caused by tick-borne viruses still impose a significant burden on affected patients.

Tonghua, Baishan, and Yanbian, situated in northeastern China, border the neighboring region of North Korea. Located within the transnational area of Changbai Mountain, these border cities are characterized by a temperate monsoon climate and is abundant in forests and wildlife resources. These environmental characteristics provide favorable conditions for tick proliferation and the circulation of tick-borne viruses [6]. TBEV has been prevalent in this region for a considerable time [7,8]. Additionally, SFTSV, one of the main tick-borne viruses in central and east China, has also been found [9]. It can cause severe fever with thrombocytopenia syndrome with a mortality rate of 10–15% [10]. Moreover, the discovery of novel tick-borne viruses, including Alongshan virus (ALSV) [11], Songling virus (SGLV) [12] and Antu virus (ATV) [13] that may pose risks for human health, was first reported in the nearby area. Prior investigations have already revealed a notable diversity of tick-borne viruses, highlighting the persistent threat posed by tick-borne diseases. Nevertheless, the continuous identification of new tick-borne viruses also suggests that routine surveillance efforts may not be adequately comprehensive. Therefore, it is crucial for additional research to obtain a deeper understanding of the ecological landscape of tick-borne viruses in this border region.

Metatranscriptomics is a powerful analytical approach that utilizes high-throughput sequencing to study the total RNA content of samples. This method offers significant advantages in terms of throughput and unbiased analysis, enabling the comprehensive analysis of pathogen information and enhancing our understanding of the virosphere. In recent years, the application of metatranscriptomics has greatly expanded our understanding of viruses, with the number of known viruses increasing from thousands to tens of thousands [14,15]. This advancement has encompassed diverse domains, including mammals [16], arthropods [17], and marine resources [18]. Furthermore, metatranscriptomics has played a crucial role in tracing the origins of the SARS-CoV-2 pandemic [19].

Transcriptomics methods have been valuable in the study of tick-borne viruses, leading to the identification of numerous new viruses [5]. While many of these newly identified viruses have not been detected in human populations, they belong to viral families capable of infecting vertebrates [5,20]. These findings have significantly updated our understanding of the diversity of viruses carried by ticks and have contributed to clinical diagnosis. A compelling example is the identification of Beiji nairovirus in questing *I. persulcatus* before it caused clinical illness in patients [21]. Therefore, the continued utilization of metatranscriptomics to explore the virome of *I. persulcatus* is a highly worthwhile effort that holds great potential for further discoveries.

In this study, we utilized metatranscriptomics to investigate the virome of *I. persulcatus* on the China–North Korea border. The object of this study was to analyze the diversity of the virome carried by *I. persulcatus* and to identify and characterize RNA viruses that have the potential to infect humans or other mammals. Additionally, we aimed to gain insights into the early ecology of newly discovered tick-borne viruses. We expect that these data provide valuable information that can contribute to disease prevention and control efforts in the China–North Korea border region. Understanding the composition and characteristics of the tick virome can assist the identification and surveillance of potential viral threats, thus enhancing preparedness and response strategies.

## 2. Materials and Methods

### 2.1. Ticks Collection and Sample Processing

**Figure 1:**
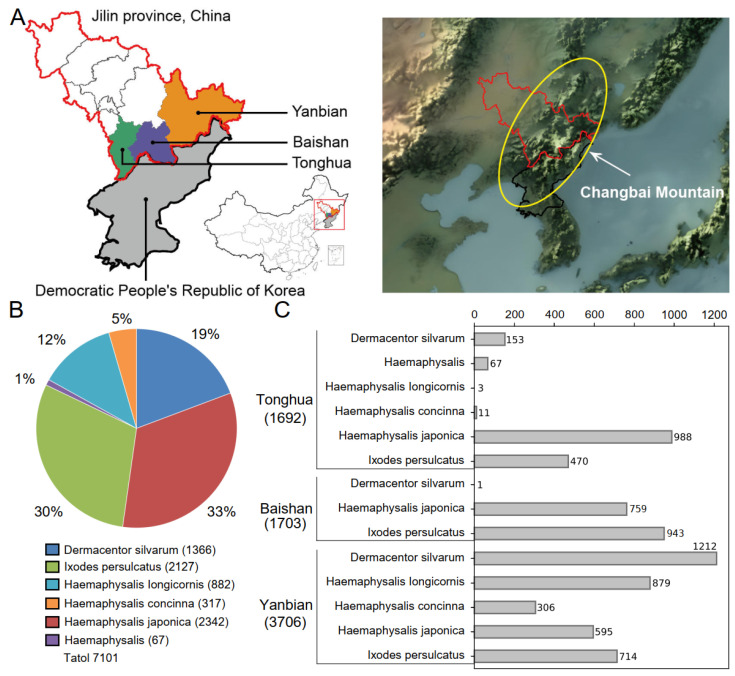
Overview of tick sample collection. (**A**) Location of tick collection (the map resource is from http://datav.aliyun.com/portal/school/atlas/area_selector (accessed on 12 April 2023), map approval number is GS Jing(2022)1061). The yellow circle in the topographic map shows the Changbai Mountain. (**B**) The number and proportion of different tick species. (**C**) Statistics of tick species collection by regions.

Between April and June 2017, comprehensive tick collection was carried out in Tonghua, Baishan, and Yanbian, located in Jilin Province, China (Figure 1A). The collection process involved gathering from various sources, including free-living ticks found in forests and grasslands, as well as ticks parasitizing on cattle and sheep. Free-living ticks were collected by dragging a flannel flag over vegetation. Parasitizing ticks were collected from animals by using tweezers. The collected ticks were then subjected to identification and classification by experienced entomologists. Approximately ten ticks of the same species were placed into a tube. The ticks were kept alive during transportation to the laboratory, then promptly stored at −80 °C for subsequent analysis and investigation.

### 2.2. RNA Extraction

To process the collected ticks, they were rinsed 3 times with PBS. Then, they were mixed with a solution of 5% penicillin and streptomycin in Minimum Essential Medium (Gibco, America). Next, the ticks were ground using a QIAGEN TissueLyserII (QIAGEN, Hilden, Germany) with 5 mm steel beads at a frequency of 25 Hz for 3 min. After grinding, the tick specimens were centrifuged at 4 °C and 20,000× *g* for 30 min. The supernatant was transferred to sterile EP tubes, ensuring that no particulate matter was transferred. Total RNA extraction and purification were carried out using a CqEx-DNA/RNA virus (CDC) kit (TIANLONG, Xi’an, China) according to the manufacturer’s instructions. The quality of the extracted nucleic acid was evaluated using a Qubit 4.0 fluorometer (Thermo Fisher, Eugene, OR, USA).

### 2.3. Library Construction, and Sequencing

The construction of RNA sequencing libraries was carried out using the VAHTS Universal V8 RNA-seq Library Prep Kit for Illumina (Vazyme, Nanjing, China). After library construction, paired-end sequencing with a read length of 150 bp was performed using the NovaSeq 6000 platform (Illumina, San Diego, CA, USA). All sequencing operations, including library loading, cluster generation, and sequencing, were conducted based on the protocols recommended by the manufacturer.

### 2.4. Virus Discovery and Viral Abundance Estimation

After sequencing, the quality of each library was evaluated using FastQC v0.11.9 [22]. To ensure high-quality data for downstream analyses, Trimmomatic 0.39 was used for quality control, including the elimination of adapters and low-quality sequences [23]. To eliminate host sequences, Bowtie2 was used in an “end-to-end” mode [24], utilizing the GenBank sequence (accession number: GCA_013358835.2). The remaining reads were de novo assembled into contigs using Megahit v1.2.9 [25]. The assembled contigs were then compared to the GenBank nr database using Diamond blastx with a threshold of 1E-5. The parameter setting of “more-sensitives” was employed to enhance sensitivity during the comparison [26]. Species annotation was performed using Megan [27]. Then, viral contigs were extracted from the assembly for further analysis. The quality of these contigs was evaluated by using Quast v5.2.0 [28]. To assess the abundance of viruses, Salmon v1.4.0 was used to quantify the transcripts per million (TPM) and read counts of the virus contigs [29]. A Python script was used to merge the abundance of the same family or species within the same library, generating viral operational taxonomic unit (vOTU) tables for further analysis and interpretation.

### 2.5. Genome Assembly

This study solely focuses on RNA viruses. The open reading frame (ORF) regions within the viral contigs were predicted by using Prokka v1.14.6 [30]. Next, the predicted ORF regions were compared to the RdRp-scan 0.90 database using Diamond blastx to gather information specifically related to RNA viruses [31]. To assemble the complete viral genomes, Bowtie2 was utilized using the corresponding reference sequence (Table S1).

To identify potential novel virus species, a threshold of <90% amino acid identity for the RNA-dependent RNA polymerase (RdRp) was utilized. RdRp-scan 0.90 and the online blast tool (https://blast.ncbi.nlm.nih.gov/Blast.cgi) were used for double confirmation and validation. To ensure accurate assembly, the reads were mapped back to the assembled contigs using Bowtie2.

### 2.6. Phylogenetic Analysis

To examine the evolutionary characteristics of tick-borne viruses, we performed phylogenetic analysis. Sequences from external sources were obtained from the NCBI database, and their sequence information was recorded (Tables S2 and S3). Multiple sequence alignment was performed using Mafft v7.450 software [32]. Genetic distances were calculated using MEGA7 [33]. Maximum likelihood (ML) trees were constructed using IQ-TREE software version 1.6.12 [34]. The most appropriate substitution models were determined using ModelFinder in IQTREE [35]. The resulting phylogenetic tree was visualized using Chiplot (www.chiplot.online (accessed on 3 July 2023)) [36].

### 2.7. Statistical Analyses and Visualization

Kruskal–Wallis test was performed using the statannotations library in Python 3.7. Alpha diversity measures, including observed richness and Shannon index, were calculated using the vegan package in R. Beta diversity analysis, employing non-metric multi-dimensional scaling (NMDS), was also performed based on the Bray–Curtis dissimilarity matrix using the vegan package in R [37]. Box plots, scatter plots, and bar charts were generated using the Seaborn library. The heatmap was created using the PyComplexHeatmap library. Stacked plots were created using the Pandas library and the venn diagram was created using the Venn library in Python 3.7.

## 3. Results

### 3.1. Overview of Sample Collection

From April to June 2017, a total of 7101 ticks were collected in Tonghua, Baishan, and Yanbian of Jilin Province, China (Table S4). These ticks belonged to five different identified species, with their proportions listed in descending order: *Haemaphysalis japonica* (ticks = 2342, 33%), *I. persulcatus* (ticks = 2127, 30%), *Dermacentor silvarum* (ticks = 1366, 19%), *Haemaphysalis iongicornis* (ticks = 882, 12%), and *Haemaphysalis concinna* (ticks = 317, 5%). There were also a small number of unidentified *Haemaphysalis* ticks (ticks = 67, 1%) (Figure 1B). Among the three regions studied, both Tonghua and Yanbian were found to have all five species of ticks, while Baishan only had three species, namely *D. silvarum*, *H. japonica*, and *I. persulcatus* (Figure 1C).

### 3.2. Overview of RNA Sequencing of I. persulcatus

In subsequent investigations, we specifically focused on *I. persulcatus*, given its potential to harbor novel viruses capable of infecting humans. To facilitate library preparation and sequencing, whole ticks of *I. persulcatus* were grouped into pools consisting of 20–75 individuals per tube. A total of 53 libraries were generated, with 13 pools from Tonghua, 19 from Baishan, and 21 from Yanbian. Each library yielded a range of 3,709,032–37,987,814 clean reads, and within these reads, there were 3213–3,380,664 viral reads (Table S5). The proportion of viral reads in the clean reads varied from 0.03% to 13.90%. Following assembly, a total of 157,766 viral contigs (>500 bp) were obtained, with an average N50 length of 1349 bp (Table S5).

### 3.3. Diversity of I. persulcatus Virome in Tonghua, Baishan and Yanbian

**Figure 2:**
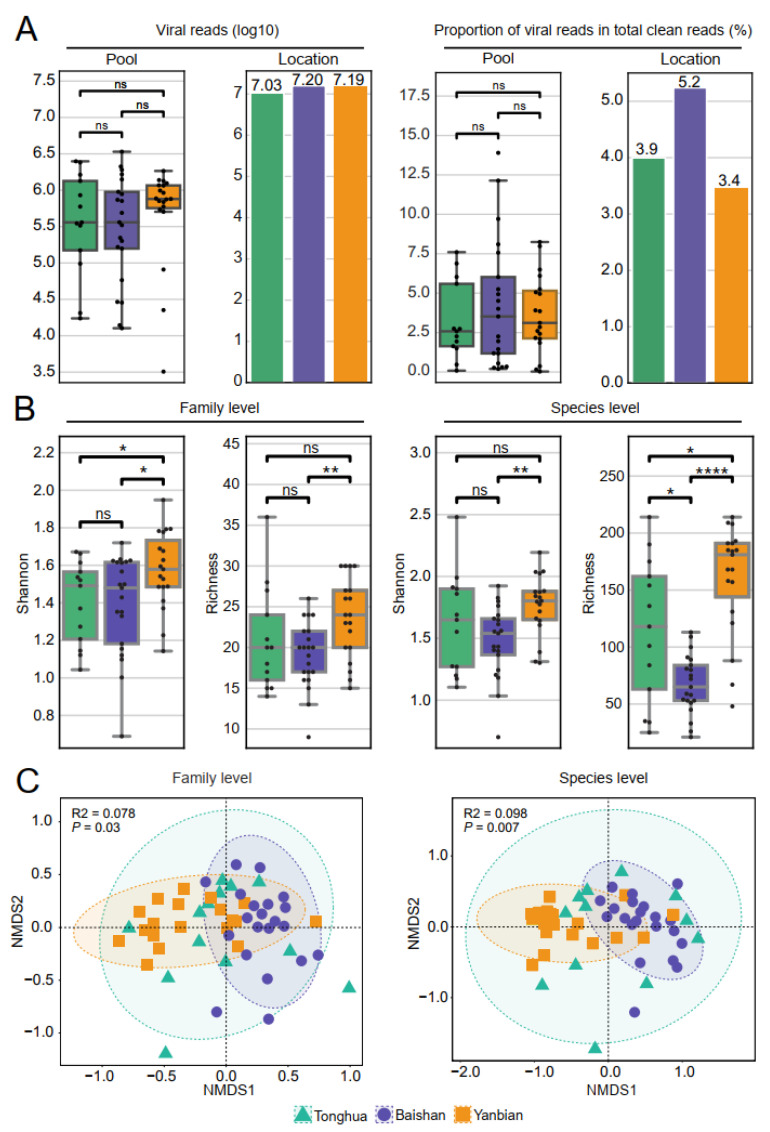
Diversity and composition of the tick virome. (**A**) Counts and proportions of virus reads in each library or region, with each dot representing one library. Alpha diversity and beta diversity analyses at family and species level based on vOTUs. (**B**) Alpha diversity analysis of virus reads from *I. persulcatus* in Tonghua, Baishan, and Yanbian. Kruskal–Wallis test: *p* < 1 (ns), *p* < 0.05 (*), *p* < 0.01 (**), *p* < 0.0001 (****). (**C**) NMDS analysis based on Bray–Curtis distance, reflecting the viral composition characteristics among the three regions. Green, purple, and orange represent Tonghua, Baishan, and Yanbian respectively.

To examine the viral load and diversity of ticks from the three regions, a comparative analysis was conducted on the viral reads. The proportion of viral reads in Baishan was slightly higher (5.2%) than in the other two areas, but the difference was not statistically significant (Kruskal–Wallis test, *p* > 0.05). The number and proportion of viral reads did not show a significant difference among the three regions (Figure 2A and Table S6).

To further examine the characteristics of virus diversity in ticks from the three regions, we performed alpha and beta diversity analysis at the family and species levels based on viral operational taxonomic units (vOTUs). The Shannon index and observed richness analysis indicated the degree of diversity within each group. Ticks from Yanbian showed significantly higher viral diversity compared to those from Baishan (Kruskal–Wallis test, *p*-values ranging from 5.695 × 10^−6^ to 3.130 × 10^−2^) at the family and species levels. The viral diversity in ticks from Yanbian was slightly higher than in ticks from Tonghua (Kruskal–Wallis test, *p*-values ranged from 2.868 × 10^−2^ to 1.987 × 10^−1^) (Figure 2B and Table S6). However, there was no significant difference in viral diversity between ticks from Baishan and Tonghua (all three indices showed *p* > 0.05). Beta diversity analysis, specifically NMDS, based on the Bray–Curtis distance was performed to visualize the differences in species composition. Ticks from Baishan and Yanbian showed distinct clustering patterns (Figure 2C). The Permutational multivariate analysis of variance (PERMANOVA) test confirmed significant differences in viral diversity between Baishan and Yanbian at both the family level (*p* < 0.05 and R^2^ = 0.09) and the species level (*p* < 0.05 and R^2^ = 0.10) (Table S6).

### 3.4. Vertebrate-Associated RNA Virus Families

**Figure 3:**
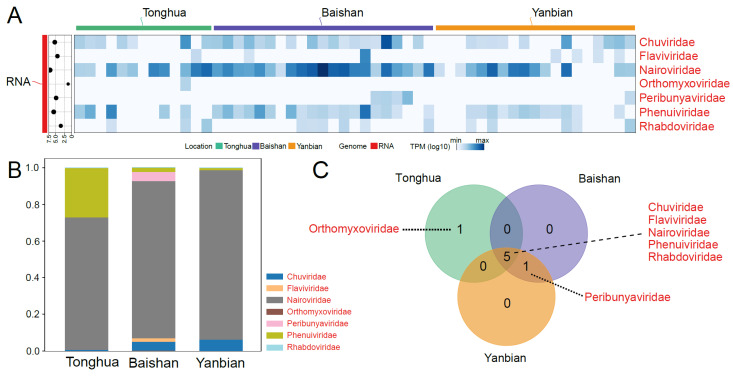
The abundance and diversity of the vertebrate-associated RNA virome. (**A**) A heatmap represents the abundance of viral families in TPM levels, while the scatter plot on the left represents the total number of reads (log10) for each viral family. The color bar indicates the region or genome type. (**B**) A stacked bar chart displays the relative abundance of viral families in three different locations. (**C**) A Venn diagram illustrates the viral families shared or unique among three different locations. The red font represents RNA viruses.

In this study, 7 RNA virus families that may infect humans or other mammals (Figure 3A) were identified among the vOTUs, including *Chuviridae*, *Flaviviridae*, *Nairoviridae*, *Orthomyxoviridae*, *Peribunyaviridae*, *Phenuiviridae*, and *Phenuiviridae*. Among these families, *Nairoviridae* showed a high abundance in all three regions (Figure 3A,B).

The Venn diagram illustrates the overlap of virus families among the three regions. Five virus families (*Chuviridae*, *Flaviviridae*, *Nairoviridae*, *Phenuiviridae*, and *Phenuiviridae*) were shared among the three regions (Figure 3C). *Peribunyaviridae* and *Orthomyxoviridae* were sporadically identified (Figure 3C). Overall, the RNA virus families with the potential to infect humans or other mammals showed consistency across the three locations along the China–North Korea border.

### 3.5. Characteristics of New Tick-Borne Viral Genome Phylogeny

The analysis based on vOTUs tends to reflect the viral environment encountered by ticks, but it may mistakenly attribute viral presence in the host’s blood to tick-borne transmission, when in reality, it may only be transient. To gain better insights into actively replicating viruses within tick hosts at the time of sampling and to identify novel viruses, we attempted whole-genome assembly for RNA viruses. A total of 121 complete or near-complete genomes of 12 viruses from 6 families were identified. For segmented viruses, we obtained at least the sequence of the gene containing RdRp.

**Figure 4:**
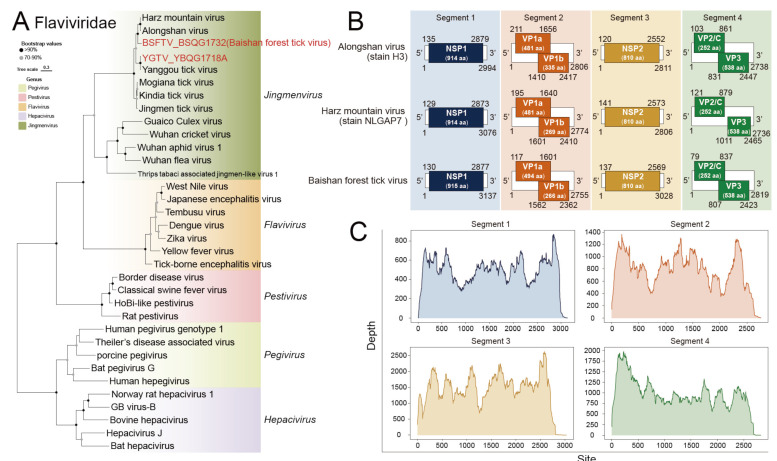
Phylogenetic analysis of the novel strains of *Flaviviridae*. (**A**) ML tree of representative viruses of *Flaviviridae* based on the RdRp region. (**B**) The genomic structure and protein sizes (sequence length in given number of amino acids (aa) per protein) of Baishan forest tick virus, Alongshan virus (strain H3), and Harz mountain virus (strain NLGAP7). (**C**) Sequencing depth plot of Baishan forest tick virus.

Among the identified viruses, we found 2 species belonging to the Jingmenvirus of *Flaviviridae* (Figure 4A). One of them was identified as the Yanggou tick virus (YGTV). The nucleotide similarities between the new Yanggou tick virus and the previously identified strain were to be 94.9–96.5% for Segment 1, 94.7–97.2% for Segment 2, 93.3–96.7% for Segment 3, and 94.5–95.9% for Segment 4 (Table S7). The other virus was considered a novel virus and tentatively named the Baishan forest tick virus (BSFTV) (Figure 4B,C). We identified four gene segments belonging to BSFTV in the sequencing data. Blast analysis revealed that these gene sequences share close phylogenetic relationships with Alongshan virus and Harz mountain virus. At the nucleotide level, the highest similarity was observed between Segment 1 of BSFTV and Segment 1 of Harz mountain virus (strain NLGAP7), with a similarity of 76.6% (Query cover 92%). For Segment 2, Segment 3, and Segment 4, BSFTV showed the closest relationship with ALSV. The similarities between BSFTV and ALSV (strain H3) were 74.7% (query cover 80%), 76.1% (query cover 89%) and 76.8% (query cover 91%).

**Figure 5:**
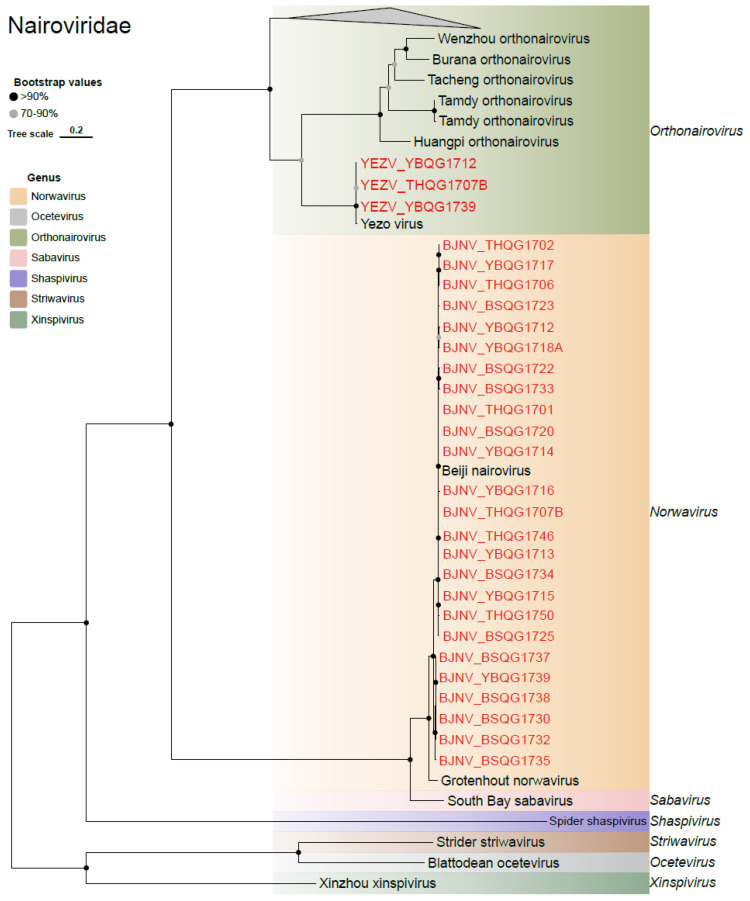
Phylogenetic analysis of the novel strains of *Nairoviridae*. ML tree of representative viruses of *Nairoviridae* based on the RdRp region.

Two viruses belonging to the *Nairoviridae* family were identified (Figure 5). One of them is the Yezo virus (YEZV), which falls under the genus Orthonairovirus. The nucleotide similarities of the S, M, and L genes of three new YEZV strains and previously identified strains ranged from 92.4 to 99.9%, 96.1 to 99.1%, and 95.1 to 99.0%, respectively (Table S7). The ML tree revealed that the three new strains belonged to different sub-lineages in the M and L genes, indicating a certain level of diversity of YEZV in the region (Figure S1). The other identified virus was Beiji nairovirus, which accounted for nearly half of the libraries with a total of 25 strains. The nucleotide similarities of the S gene and L gene between the new strain and previously identified strains were 94.9% to 99.3% and 99.16% to 99.9%, respectively (Table S7). In the ML tree, these newly discovered viruses and previous Beiji nairoviruses formed two distinct branches in the S segment, but were intermixed in the L segment (Figure S1).

**Figure 6:**
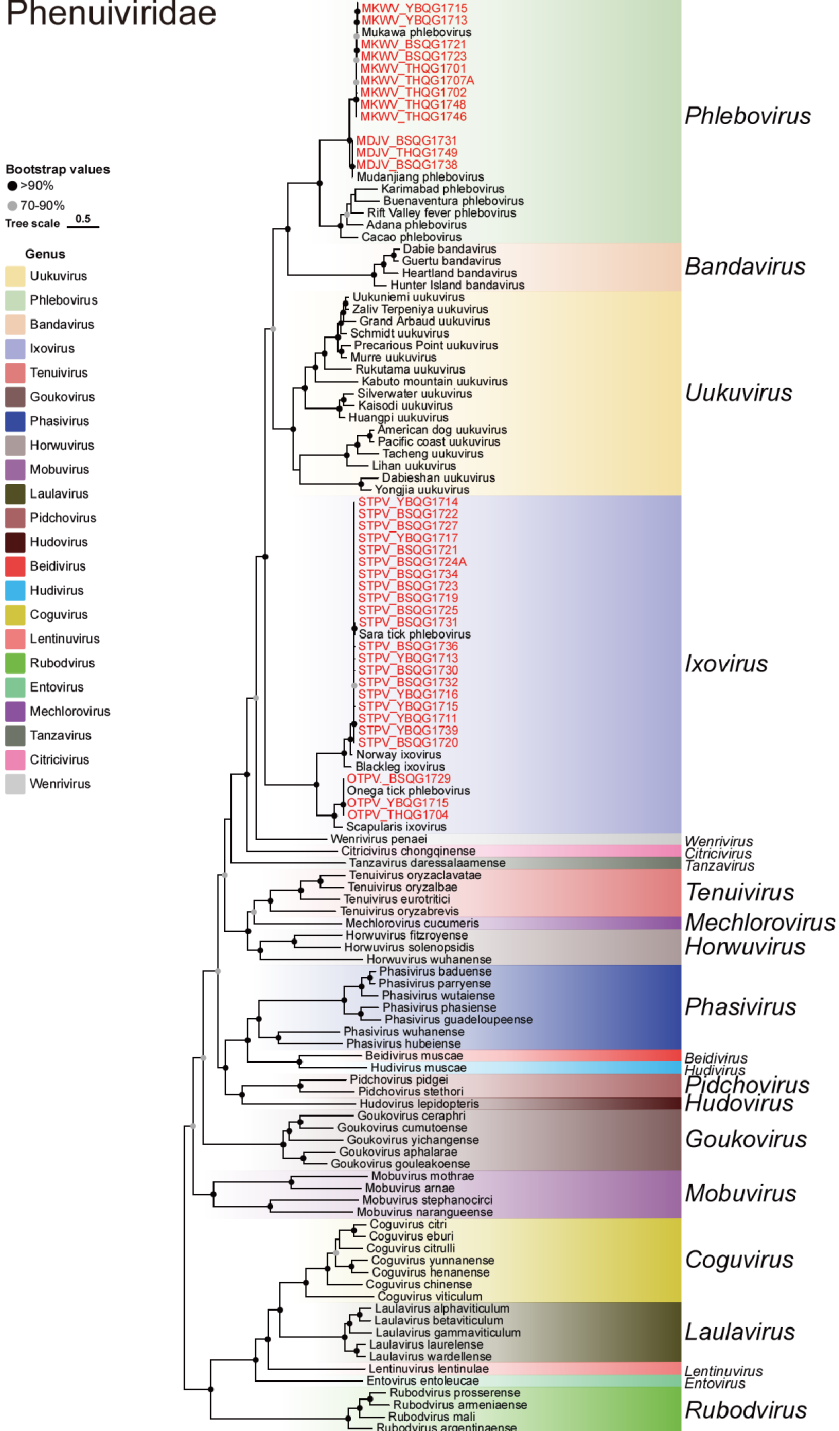
Phylogenetic analysis of the novel strains of *Phenuiviridae*. ML tree of representative viruses of *Phenuiviridae* based on the RdRp region.

Four viruses belonging to the *Phenuiviridae* family were identified, namely Sara tick phlebovirus (STPV), Onega tick phlebovirus (OTPV), Mukawa virus (MKWV), and Mudanjiang phlebovirus (MDJV) (Figure 6). STPV was first detected in *I. persulcatus* ticks collected in 2018 (Table 1). This study identified 20 new strains of STPV, accounting for 37.7% (20/53) of the total libraries. The nucleotide similarity of the S gene between these viruses and previously identified strains was greater than 99.0%, while the nucleotide similarity of the L gene ranged from 92.9% to 99.9% (Table S7). Moreover, these new strains clustered together with the previous strains without forming new lineages, indicating a close relationship between the strains from 2017 and the previously discovered ones. Nine new MKWV strains were detected, bridging the gap between previously distant strains and suggesting the crucial role of MKWV in viral evolution in this region. The nucleotide similarities of the S, M, and L genes between the new and previous strains of MKWV were 93.2–97.3%, 94.5–99.9%, and 91.8–96.8%, respectively (Table S7). OTPV and MDJV were newly discovered viruses detected in *I. persulcatus* samples in 2018 and 2021, respectively (Table 1). In this study, three new strains of OTPV and three new strains of MDJV were identified in *I. persulcatus* samples from 2017, indicating the early presence of these two viruses. The nucleotide similarities between the new OTPVs and the previous strains in the S gene and the L gene were 98.3–99.8% and 98.3–99.9%, respectively (Table S7). The nucleotide similarities between the new MDJVs and the previous strains in the S gene, M gene, and L gene were 95.4–99.2%, 99.6%, and 94.9–99.0%, respectively (Table S7).

**Figure 7:**
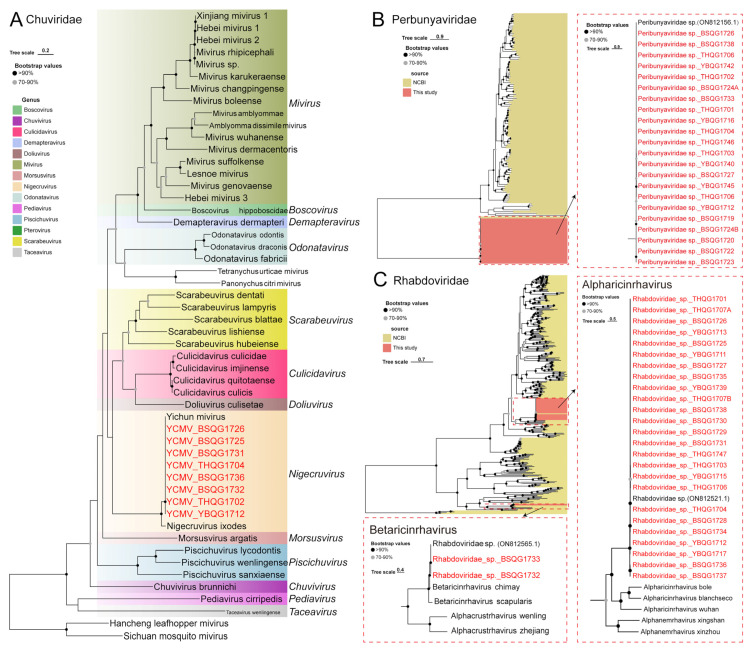
Phylogenetic analysis of the novel strains of *Chuviridae*, *Perbunyaviridae* and *Rhabdoviridae*. (**A**) ML tree of representative viruses of *Chuviridae* based on the RdRp region, (**B**) ML tree of representative viruses of *Perbunyaviridae* based on the RdRp region, (**C**) ML tree of representative viruses of *Rhabdoviridae* based on the RdRp region.

Yichun mivirus (YCMV), a virus belonging to the *Nigecruvirus* genus of *Chuviridae*, was detected in the sequencing data (Figure 7A). YCMV was initially discovered in *I. persulcatus* ticks in Northeast China in 2020 (Table 1). In this study, eight new strains of YCMV were identified in the *I. persulcatus* samples from 2017, indicating the presence of this virus in the region even earlier (Figure S2A). The nucleotide similarity between these new strains and the previously identified strains ranged from 98.9% to 99.9% at the whole-genome level (Figure S2A).

A large number of Peribunyaviridae-like viruses were also found, with complete L gene sequences of 21 strains. These viruses exhibited high similarity with Peribunyaviridae sp. (ON812156.1) previously detected in ticks in 2019 (Figure 7B). The nucleotide identity of the L genes among this group of viruses was greater than 99.0% (Figure S2B). Although we adopted the previous classification, the phylogenetic tree suggests that these new viruses are more like an outgroup of *Peribunyaviridae* (Figure S2B).

The sequencing data revealed the presence of two unnamed viruses belonging to *Rhabdoviridae* family, specifically 25 strains of Alpharicinrhavirus and 2 strains of Betaricinrhavirus (Figure 7C). These two viruses were already detected in samples from 2019 (Table 1). In *Alpharicinrhavirus*, the nucleotide similarity between the new strains identified in this study and the previously discovered Rhabdoviridae sp. virus from 2019 ranged from 95.8% to 99.9% at the whole-genome level (Figure S8C). As for *Betaricinrhavirus*, the new strains identified in this study exhibited nucleotide similarity greater than 99.0% with the newly discovered Rhabdoviridae sp. virus in 2019 at the whole-genome level (Figure S8D).

## 4. Discussion

From April to June 2017, an extensive tick collection was carried out in the border regions between China and North Korea, with a specific focus on Tonghua, Baishan, and Yanbian. Employing transcriptome sequencing, we thoroughly characterized the viral composition of *I. persulcatus*. The study encompassed 2127 samples of *I. persulcatus* ticks, revealing a high abundance and diversity of viruses carried by ticks. Interestingly, we observed differences in the tick-borne virus spectrum among ticks from Tonghua, Baishan, and Yanbian, despite their geographic proximity. Furthermore, our findings suggest that the presence of some emerging tick-borne viruses in northeast China is not isolated but rather commonly found within the tick ecological circle. Through phylogenetic analysis, we discovered that certain viruses in the region play a crucial role in viral evolution and transmission. Importantly, this study extends the earliest known presence of seven emerging tick-borne viruses back to 2017, including MDJV, OTPV, STPV, YCMV and three unnamed viruses from the *Peribunyaviridae* and *Phenuiviridae* families.

While differences were identified in the *I. persulcatus* virome among different regions, no apparent distinction was found for viruses with the potential to infect humans. Although previous metagenomic studies have explored the tick-borne pathogen spectrum in the region [5,6], we provided a more comprehensive understanding of tick-borne virus diversity across the three regions. Alpha and beta diversity analyses revealed that Yanbian shows significantly greater diversity at the viral family and species levels compared to Baishan, and the viral spectrum composition of *I. persulcatus* also differs between these two regions. Conversely, the geographical characteristics of the tick-borne virus spectrum in Tonghua were less evident. Geographical variations in virus abundance and diversity have also been observed in studies on tick-borne virus diversity in Xinjiang and Inner Mongolia [38]. As ticks themselves lack the ability for long-distance transmission, the viruses they carry are likely acquired from local organisms that they come into contact with. The observed geographical differences in viral spectra encompass viral pathogens in animals, plants, bacteria, and fungi. Notably, the three locations shared most of the known viral families capable of infecting humans, which is expected given the smaller evolutionary divergence between mammals and arthropods compared to other organisms. Overall, administrative regional divisions have not overridden the shaping influence of natural geography when it comes to natural human threats.

In this study, we detected two emerging tick-borne viruses associated with human febrile illnesses [21,39], namely the Yezo virus and the Beiji nairovirus. Fever cases related to Yezo virus have been reported in Japan and China, with YEZV antibodies also detected in wild deer and raccoons in Hokkaido, Japan [39,40]. Currently, YEZV nucleic acid has only been detected in *I. persulcatus* in China, with a positivity rate of approximately 0.5% [39]. In Japan, YEZV nucleic acid has only been detected in *H. megaspinosa*, *I. ovatus*, and *I. persulcatus*, with an overall positivity rate of 2.1% [40]. Although our study only identified three new YEZV strains with a low positive rate, it is important to note that our pooled sequencing cannot determine the individual tick-level positivity rate. Beiji nairovirus has exclusively been found in northeastern China, with the earliest detection dating back to 2015 in *I. persulcatus* [6,21,41]. In our study, Beiji nairovirus was detected in 25 out of 53 libraries, indicating a relatively high positivity rate. Our findings demonstrate the presence of the Yezo virus and Beiji nairovirus in the China–North Korea border area in 2017, and these viruses from 2017 share a close phylogenetic relationship with the recently discovered Yezo virus (Heilongjiang, Inner Mongolia) and Beiji nairovirus in northeastern China. This suggests that these viruses have likely established stable circulation cycles and may continue to spread in the future. Consequently, it is important for regional managers to be attentive to the diseases that may be caused by these two viruses.

An important finding of our study is the discovery of the novel Baishan forest tick virus, which belongs to *Flaviviridae* family, and Jingmenviruses genus. BSFTV shows a close genetic relationship with the Alongshan virus and the Harz mountain virus. Typically, Jingmenviruse is known to have four- or five-segment genes. For instance, the Alongshan virus and Harz mountain virus are four-segment viruses [42,43], while the Guaico Culex virus has five segments [44]. In our sequencing libraries, we recovered four segments of the Baishan forest tick virus. It is important to note that virus isolation experiments are the gold standard for confirming the existence of a real virus. However, as of the publication of this article, such experiments have not been successful. Nevertheless, this does not diminish the significance of highlighting the importance of this virus, because Alongshan virus is considered to be associated with febrile illnesses [9], and serological investigations have found ALSV-specific antibodies in sheep and cattle [45]. Therefore, it is possible that BSFTV, which is closely related to ALSV, may also be associated with mammalian diseases. While there is currently insufficient evidence to directly link these viruses to human diseases, it is worth noting that RNA viruses, particularly segmented RNA viruses, can quickly acquire new viral features through mutation or reassortment [46]. As a result, we should remain vigilant regarding the potential evolution of these viruses within the tick population and the future threats they may pose.

There are additional noteworthy phenomena that deserve attention in this study. Consistent with previous studies, we observed that newly tick-borne viruses in China were closely related to strains found in Japan [47]. We also identified some viruses, such as YGTV, that were closely related to Russian strains. The phenomenon of long-distance virus transmission has already been observed, as exemplified by TBEV. TBEV is known to comprise three primary subtypes, each associated with distinct geographical regions: European, Siberian, and Far East. These subtypes have the capacity to induce varying clinical presentations in humans, each with varying morbidity and mortality rates [48]. Furthermore, recent research has unveiled two additional subtypes: the Baikalian subtype, comprising 13 distinct strains identified in eastern Siberia and northern Mongolia, and the Himalayan subtype, isolated in rodents from the Qinghai–Tibet Plateau region of China [49,50]. This raises the possibility of similar scenarios occurring in other tick-borne viruses.

Migratory birds are considered one of the pathways for the long-distance transmission of vector-borne viruses [51,52]. The migration route of birds from Japan to Northeast China passes through South Korea and North Korea, while the path from Russia to Northeast China passes through Mongolia [53]. As a consequence, it is conceivable that these viruses may have already expanded their range to intermediary countries, potentially establishing viral reservoirs within these regions. However, definitive evidence would require the detection of the virus within bird samples.

There are certain limitations in this study. We were not able to identify some well-known tick-borne viruses, such as TEBV, SFTSV, and Nairobi sheep disease virus. Moreover, there are other species of tick present in the region, such as *D. silvarum*, *H. concinna*, *H. japonica*, and *H. iongicornis*. Although we collected tick samples from these species, we did not perform metatranscriptomic sequencing or qRT-PCR testing on them. Undoubtedly, conducting transcriptomic analysis on a wider range of tick samples would provide insights into the spectrum of tick-borne viruses and help us understand the cycling of viruses among ticks, as well as uncover tick species-specific tick-borne viruses. Furthermore, our study did not explore the viral differences between unfed and feeding ticks. This will be the focus of our future work.

Furthermore, the results of metatranscriptomic studies require further experimental validation to accurately reflect the existence and practical threat of the viruses. Additionally, natural environmental changes are influencing tick habitats, which in turn affects the ecology of tick-borne viruses [54]. Therefore, further studies should record the environmental factors in tick activity areas, enabling the use of advanced methods such as mathematical models or machine learning to enhance our understanding of virus transmission [55,56].

## 5. Conclusions

In conclusion, this study examined the viral spectrum characteristics of *I. persulcatus* at the China–North Korea border, shedding light on the evolutionary history and potential risks associated with certain viruses. The findings provide valuable reference data for the prevention and control of tick-borne diseases in this region. It is important to note that the emergence of some viruses may have occurred earlier than what was identified in this study. Therefore, it is critical to enhance the monitoring of tick-borne viruses and conduct high-throughput sequencing on tick samples from previous years to better understand the historical ecology of tick-borne viruses. These efforts will support a more comprehensive understanding of the threats posed by tick-borne viruses and aid the development of effective management strategies.

## Figures and Tables

**Table 1:** Records of virus reporting and host history before this study *.

Virus Species	Earliest Sample Time and Country	Host
Beiji nairovirus	2015 (China)	1. *I. persulcatus* 2. *Homo sapiens*
Mukawa virus	2013 (Japan)	1. *I. persulcatus* 2. *H. concinna* 3. *D. silvarum*
Mudanjiang phlebovirus ^#^	2021 (China)	1. *I. persulcatus*
Onega tick phlebovirus ^#^	2018 (Russia)	1. *I. persulcatus*
*Peribunyaviridae* sp. (ON812156.1) ^#^	2019 (China)	1. *I. persulcatus*
*Rhabdoviridae* sp. (ON812482.1) ^#^	2019 (China)	1. *I. persulcatus*
*Rhabdoviridae* sp. (ON812565.1) ^#^	2019 (China)	1. *I. persulcatus*
Sara tick phlebovirus ^#^	2018 (Russia)	1. *I. persulcatus*
Yanggou tick virus	2014 (China)	1. *D. reticulatus* 2. *I. persulcatus*
Yezo virus	2016 (Japan)	1. *I. persulcatus* 2. *H. sapiens*
Yichun mivirus ^#^	2020 (China)	1. *I. persulcatus*

*: The data was provided by the NCBI virus database. ^#^: This study found early strain of the virus.

## Data Availability

All the data generated during the current study are included in the manuscript and/or the Supplementary Data. All sequencing reads have been deposited in the CNCB databases (www.cncb.ac.cn, access on 1 September 2023) under the GSA accession CRA011751. Relevant virus genome sequences have been deposited in the GenBase databases (accession: C_AA030968.1-C_AA031172.1). Additional data related to this article may be requested from the corresponding authors.

## Supplementary Materials

The following supporting information can be downloaded at: https://www.mdpi.com/article/10.3390/v16010062/s1, Table S1: Reference sequences used for virus genome assembly. Table S2: GenBank accession numbers of RdRp sequences of reconstructing ML trees. Table S3: GenBank accession numbers of sequences used for reconstructing ML trees, similarity analysis, and collecting report time, location, and hosts information for each virus. Table S4: Sample information. Table S5: Details of sequencing data. Table S6: Raw results of statistical analysis. Table S7: The results of genetic distance analysis for each virus. Figure S1: ML trees for each virus found in this study. Figure S2: Similarity matrix of the Yichun mivirus, the *Peribunyaviridae* sp. and the *Rhabdoviridae* sp. of Alpharicinrhavirus and Betaricinrhavirus sorted by viral isolation time.
