# Supplementary material for: Metatranscriptomics Reveals the RNA Virome of Ixodes Persulcatus in the China–North Korea Border, 2017
Source: Viruses. 2023 Dec 29;16(1):62. doi: 10.3390/v16010062 (PMC10819109; doi:10.3390/v16010062)
Supplement: Supplementary file 1 [file viruses-16-00062-s001.zip › Figure S1.pdf]

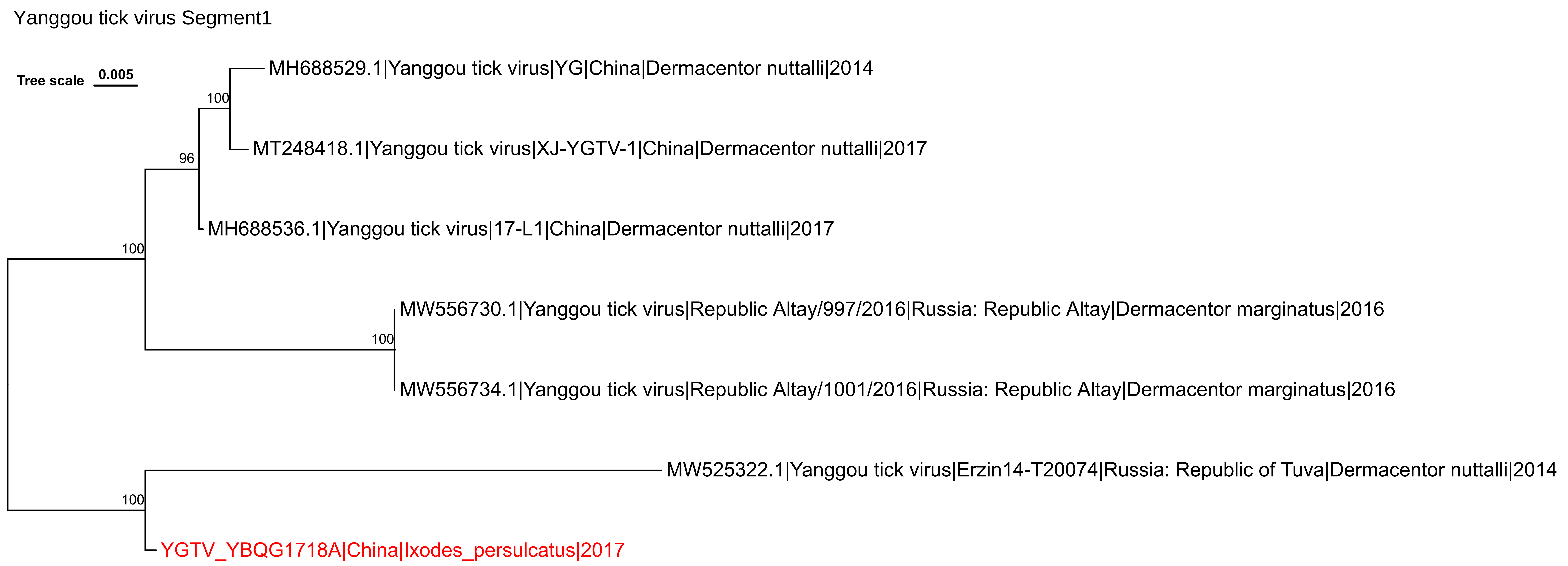
Yanggou tick virus Segment1
Tree scale
0.005
MH688529.1|Yanggou tick virus|YG|China|Dermacentor nuttalli|2014
100
MT248418.1|Yanggou tick virus|XJ-YGTV-1|China|Dermacentor nuttalli|2017
96
MH688536.1|Yanggou tick virus|17-L1|China|Dermacentor nuttalli|2017
100
MW556730.1|Yanggou tick virus|Republic Altay/997/2016|Russia: Republic Altay|Dermacentor marginatus|2016
100
MW556734.1|Yanggou tick virus|Republic Altay/1001/2016|Russia: Republic Altay|Dermacentor marginatus|2016
MW525322.1|Yanggou tick virus|Erzin14-T20074|Russia: Republic of Tuva|Dermacentor nuttalli|2014
100
YGTV_YBQG1718A|China|Ixodes_persulcatus|2017

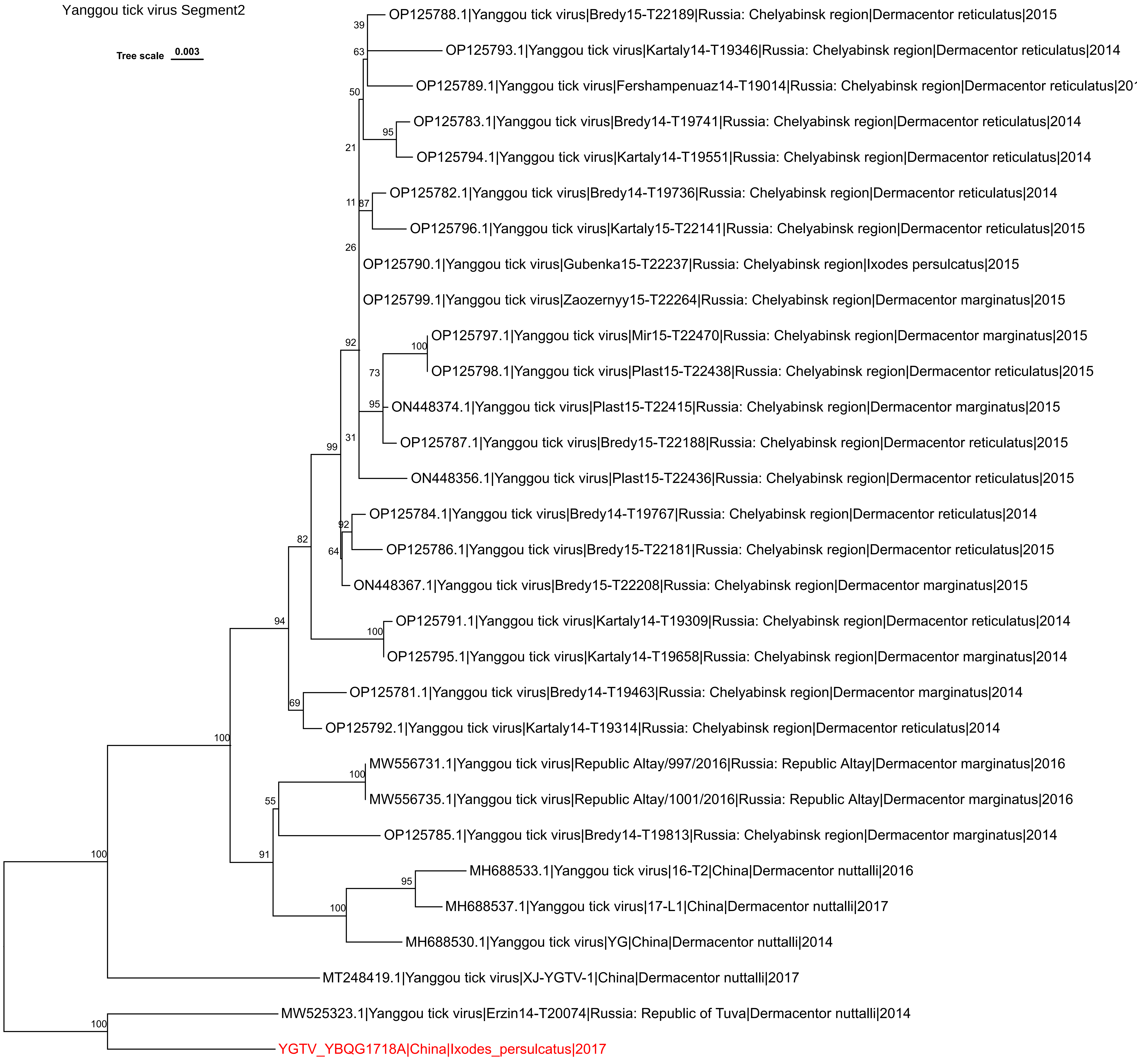
Yanggou tick virus Segment2
Tree scale 0.003
OP125788.1|Yanggou tick virus|Bredy15-T22189|Russia: Chelyabinsk region|Dermacentor reticulatus|2015
OP125793.1|Yanggou tick virus|Kartaly14-T19346|Russia: Chelyabinsk region|Dermacentor reticulatus|2014
OP125789.1|Yanggou tick virus|Fershampenuaz14-T19014|Russia: Chelyabinsk region|Dermacentor reticulatus|201
OP125783.1|Yanggou tick virus|Bredy14-T19741|Russia: Chelyabinsk region|Dermacentor reticulatus|2014
OP125794.1|Yanggou tick virus|Kartaly14-T19551|Russia: Chelyabinsk region|Dermacentor reticulatus|2014
OP125782.1|Yanggou tick virus|Bredy14-T19736|Russia: Chelyabinsk region|Dermacentor reticulatus|2014
OP125796.1|Yanggou tick virus|Kartaly15-T22141|Russia: Chelyabinsk region|Dermacentor reticulatus|2015
OP125790.1|Yanggou tick virus|Gubenka15-T22237|Russia: Chelyabinsk region|Ixodes persulcatus|2015
OP125799.1|Yanggou tick virus|Zaozernyy15-T22264|Russia: Chelyabinsk region|Dermacentor marginatus|2015
OP125797.1|Yanggou tick virus|Mir15-T22470|Russia: Chelyabinsk region|Dermacentor marginatus|2015
OP125798.1|Yanggou tick virus|Plast15-T22438|Russia: Chelyabinsk region|Dermacentor reticulatus|2015
ON448374.1|Yanggou tick virus|Plast15-T22415|Russia: Chelyabinsk region|Dermacentor marginatus|2015
OP125787.1|Yanggou tick virus|Bredy15-T22188|Russia: Chelyabinsk region|Dermacentor reticulatus|2015
ON448356.1|Yanggou tick virus|Plast15-T22436|Russia: Chelyabinsk region|Dermacentor reticulatus|2015
OP125784.1|Yanggou tick virus|Bredy14-T19767|Russia: Chelyabinsk region|Dermacentor reticulatus|2014
OP125786.1|Yanggou tick virus|Bredy15-T22181|Russia: Chelyabinsk region|Dermacentor reticulatus|2015
ON448367.1|Yanggou tick virus|Bredy15-T22208|Russia: Chelyabinsk region|Dermacentor marginatus|2015
OP125791.1|Yanggou tick virus|Kartaly14-T19309|Russia: Chelyabinsk region|Dermacentor reticulatus|2014
OP125795.1|Yanggou tick virus|Kartaly14-T19658|Russia: Chelyabinsk region|Dermacentor marginatus|2014
OP125781.1|Yanggou tick virus|Bredy14-T19463|Russia: Chelyabinsk region|Dermacentor marginatus|2014
OP125792.1|Yanggou tick virus|Kartaly14-T19314|Russia: Chelyabinsk region|Dermacentor reticulatus|2014
MW556731.1|Yanggou tick virus|Republic Altay/997/2016|Russia: Republic Altay|Dermacentor marginatus|2016
MW556735.1|Yanggou tick virus|Republic Altay/1001/2016|Russia: Republic Altay|Dermacentor marginatus|2016
OP125785.1|Yanggou tick virus|Bredy14-T19813|Russia: Chelyabinsk region|Dermacentor marginatus|2014
MH688533.1|Yanggou tick virus|16-T2|China|Dermacentor nuttalli|2016
MH688537.1|Yanggou tick virus|17-L1|China|Dermacentor nuttalli|2017
MH688530.1|Yanggou tick virus|YG|China|Dermacentor nuttalli|2014
MT248419.1|Yanggou tick virus|XJ-YGTV-1|China|Dermacentor nuttalli|2017
MW525323.1|Yanggou tick virus|Erzin14-T20074|Russia: Republic of Tuva|Dermacentor nuttalli|2014
YGTV_YBQG1718A|China|Ixodes_persulcatus|2017

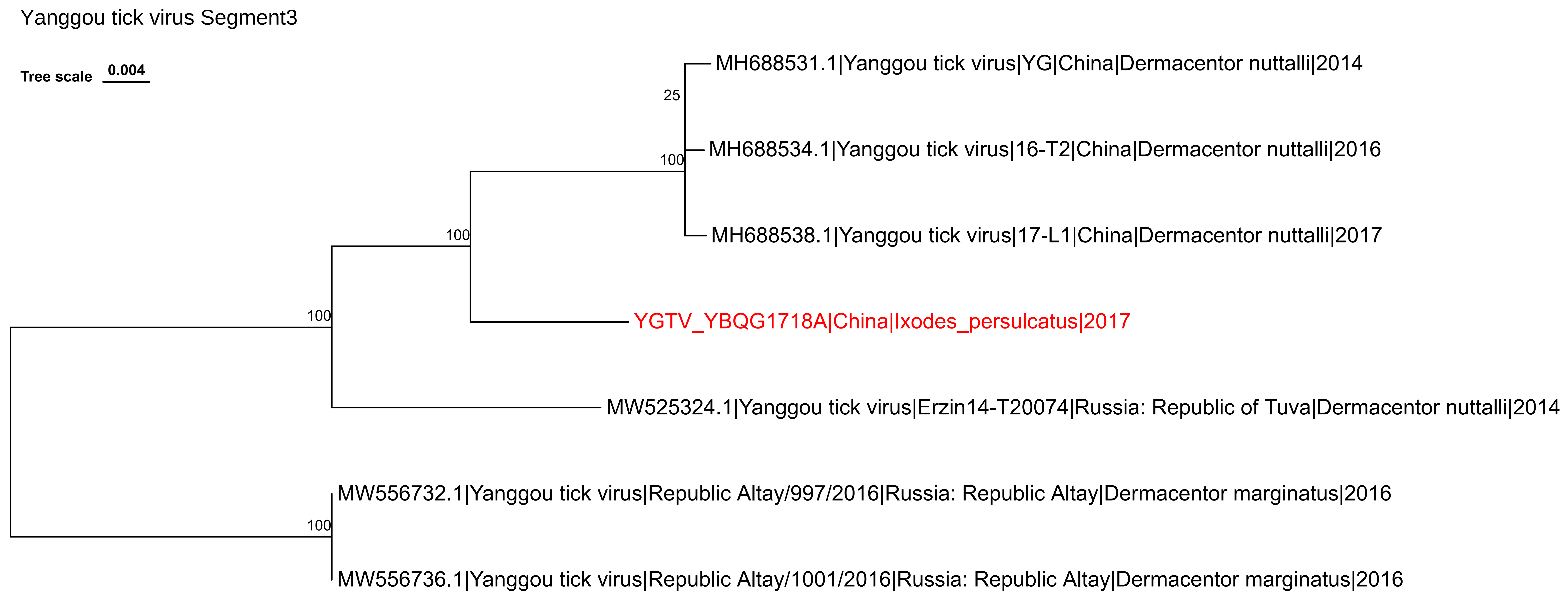
Yanggou tick virus Segment3
Tree scale
0.004
MH688531.1|Yanggou tick virus|YG|China|Dermacentor nuttalli|2014
25
MH688534.1|Yanggou tick virus|16-T2|China|Dermacentor nuttalli|2016
100
MH688538.1|Yanggou tick virus|17-L1|China|Dermacentor nuttalli|2017
100
YGTV_YBQG1718A|China|Ixodes_persulcatus|2017
100
MW525324.1|Yanggou tick virus|Erzin14-T20074|Russia: Republic of Tuva|Dermacentor nuttalli|2014
MW556732.1|Yanggou tick virus|Republic Altay/997/2016|Russia: Republic Altay|Dermacentor marginatus|2016
100
MW556736.1|Yanggou tick virus|Republic Altay/1001/2016|Russia: Republic Altay|Dermacentor marginatus|2016

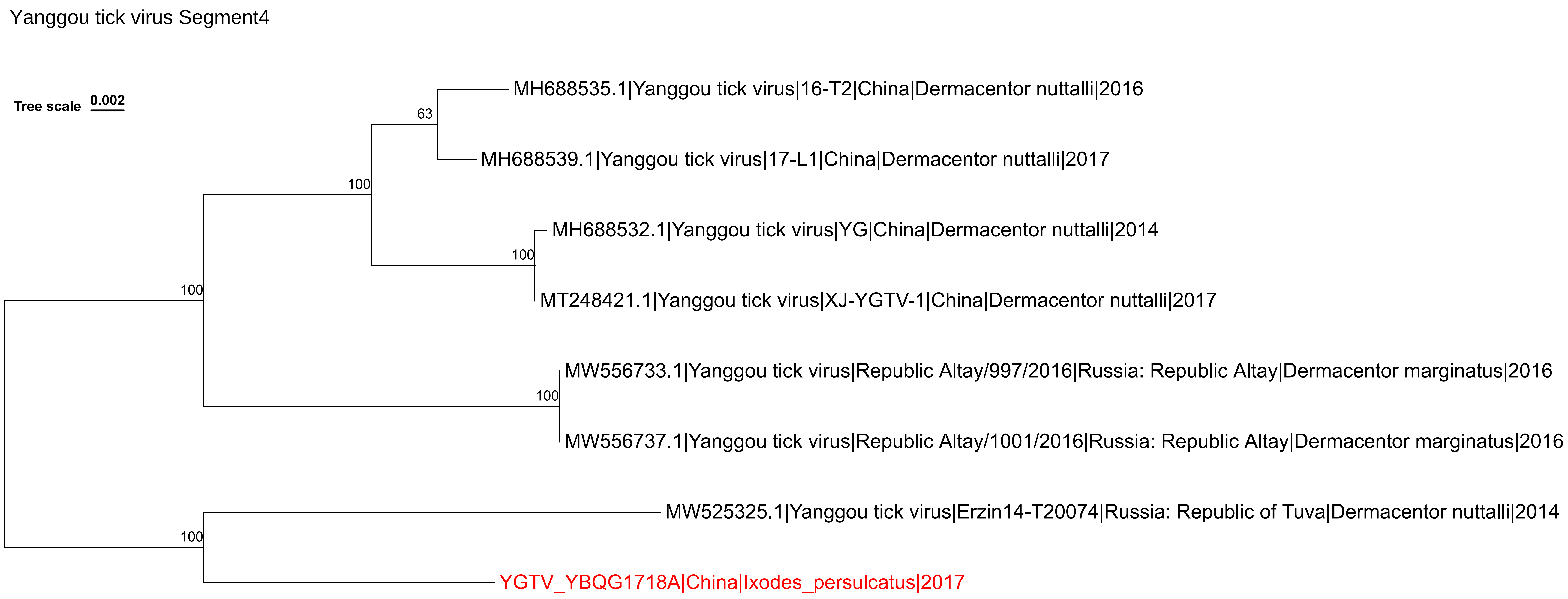

Yanggou tick virus Segment4
Tree scale 0.002
MH688535.1|Yanggou tick virus|16-T2|China|Dermacentor nuttalli|2016
63
MH688539.1|Yanggou tick virus|17-L1|China|Dermacentor nuttalli|2017
100
MH688532.1|Yanggou tick virus|YG|China|Dermacentor nuttalli|2014
100
MT248421.1|Yanggou tick virus|XJ-YGTV-1|China|Dermacentor nuttalli|2017
100
MW556733.1|Yanggou tick virus|Republic Altay/997/2016|Russia: Republic Altay|Dermacentor marginatus|2016
100
MW556737.1|Yanggou tick virus|Republic Altay/1001/2016|Russia: Republic Altay|Dermacentor marginatus|2016
MW525325.1|Yanggou tick virus|Erzin14-T20074|Russia: Republic of Tuva|Dermacentor nuttalli|2014
100
YGTV_YBQG1718A|China|Ixodes_persulcatus|2017

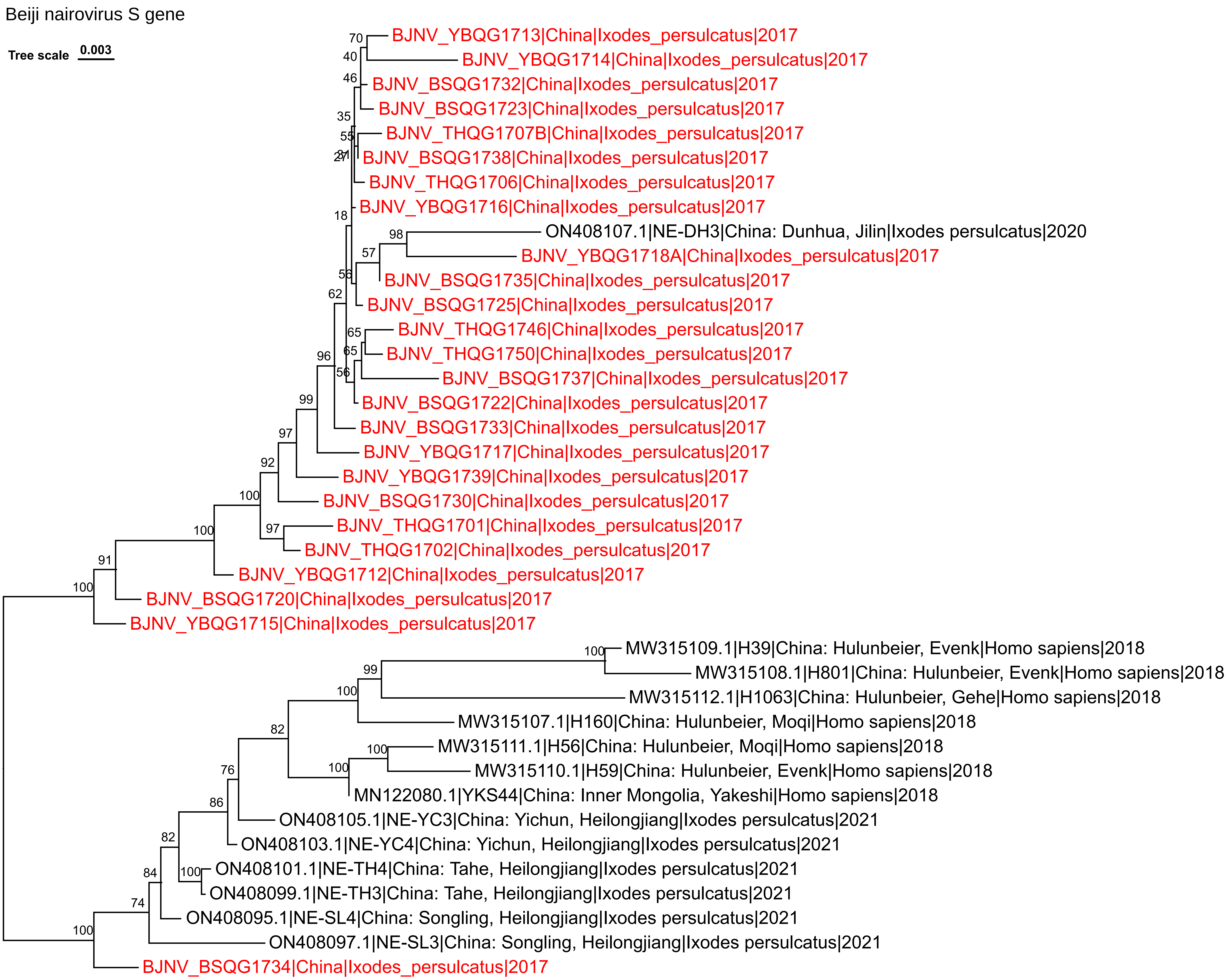

Beiji nairovirus S gene
Tree scale 0.003
BJNV_YBQG1713|China|Ixodes_persulcatus|2017
BJNV_YBQG1714|China|Ixodes_persulcatus|2017
BJNV_BSQG1732|China|Ixodes_persulcatus|2017
BJNV_BSQG1723|China|Ixodes_persulcatus|2017
BJNV_THQG1707B|China|Ixodes_persulcatus|2017
BJNV_BSQG1738|China|Ixodes_persulcatus|2017
BJNV_THQG1706|China|Ixodes_persulcatus|2017
BJNV_YBQG1716|China|Ixodes_persulcatus|2017
ON408107.1|NE-DH3|China: Dunhua, Jilin|Ixodes persulcatus|2020
BJNV_YBQG1718A|China|Ixodes_persulcatus|2017
BJNV_BSQG1735|China|Ixodes_persulcatus|2017
BJNV_BSQG1725|China|Ixodes_persulcatus|2017
BJNV_THQG1746|China|Ixodes_persulcatus|2017
BJNV_THQG1750|China|Ixodes_persulcatus|2017
BJNV_BSQG1737|China|Ixodes_persulcatus|2017
BJNV_BSQG1722|China|Ixodes_persulcatus|2017
BJNV_BSQG1733|China|Ixodes_persulcatus|2017
BJNV_YBQG1717|China|Ixodes_persulcatus|2017
BJNV_YBQG1739|China|Ixodes_persulcatus|2017
BJNV_BSQG1730|China|Ixodes_persulcatus|2017
BJNV_THQG1701|China|Ixodes_persulcatus|2017
BJNV_THQG1702|China|Ixodes_persulcatus|2017
BJNV_YBQG1712|China|Ixodes_persulcatus|2017
BJNV_BSQG1720|China|Ixodes_persulcatus|2017
BJNV_YBQG1715|China|Ixodes_persulcatus|2017
MW315109.1|H39|China: Hulunbeier, Evenk|Homo sapiens|2018
MW315108.1|H801|China: Hulunbeier, Evenk|Homo sapiens|2018
MW315112.1|H1063|China: Hulunbeier, Gehe|Homo sapiens|2018
MW315107.1|H160|China: Hulunbeier, Moqi|Homo sapiens|2018
MW315111.1|H56|China: Hulunbeier, Moqi|Homo sapiens|2018
MW315110.1|H59|China: Hulunbeier, Evenk|Homo sapiens|2018
MN122080.1|YKS44|China: Inner Mongolia, Yakeshi|Homo sapiens|2018
ON408105.1|NE-YC3|China: Yichun, Heilongjiang|Ixodes persulcatus|2021
ON408103.1|NE-YC4|China: Yichun, Heilongjiang|Ixodes persulcatus|2021
ON408101.1|NE-TH4|China: Tahe, Heilongjiang|Ixodes persulcatus|2021
ON408099.1|NE-TH3|China: Tahe, Heilongjiang|Ixodes persulcatus|2021
ON408095.1|NE-SL4|China: Songling, Heilongjiang|Ixodes persulcatus|2021
ON408097.1|NE-SL3|China: Songling, Heilongjiang|Ixodes persulcatus|2021
BJNV_BSQG1734|China|Ixodes_persulcatus|2017

Beiji nairovirus L gene

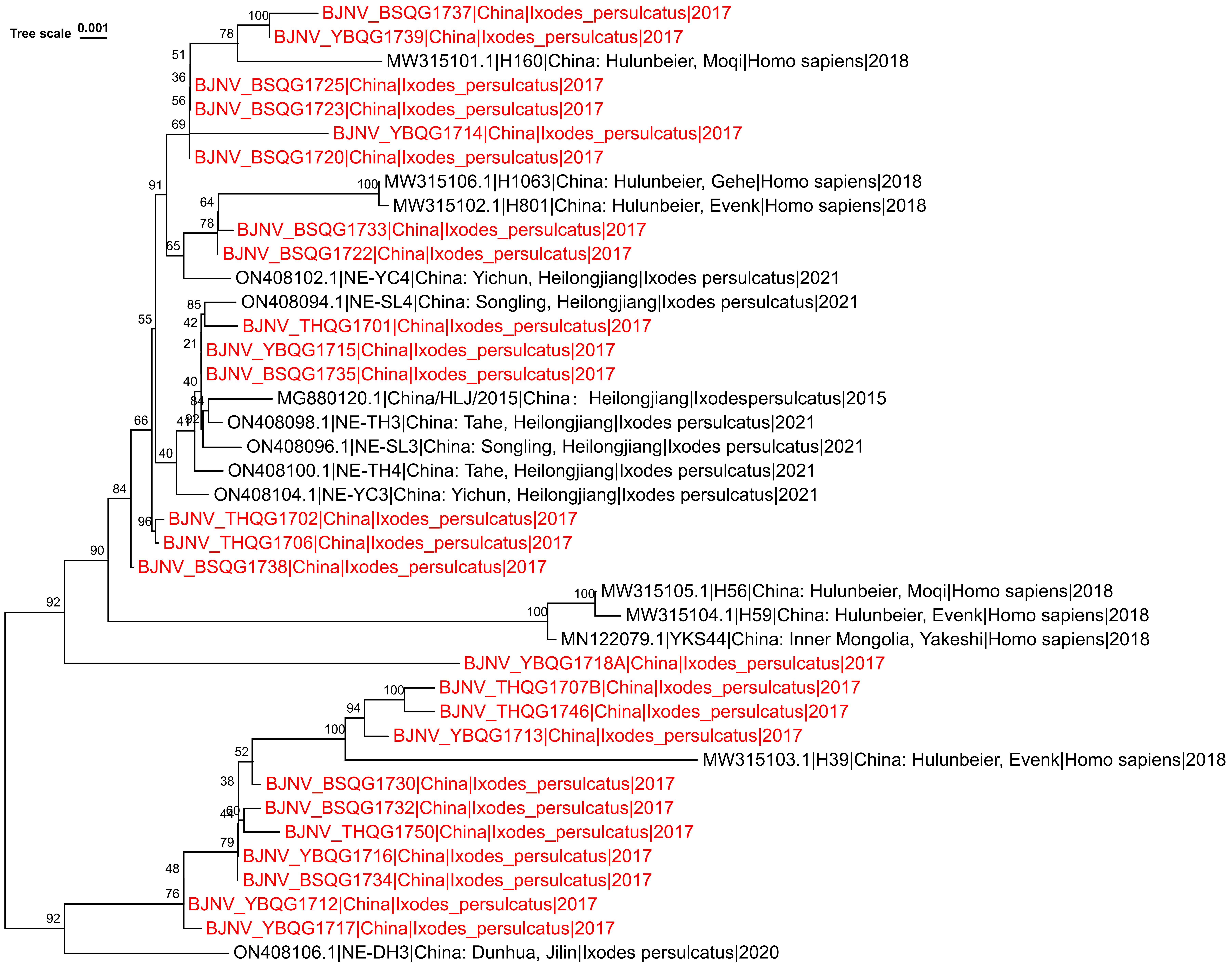

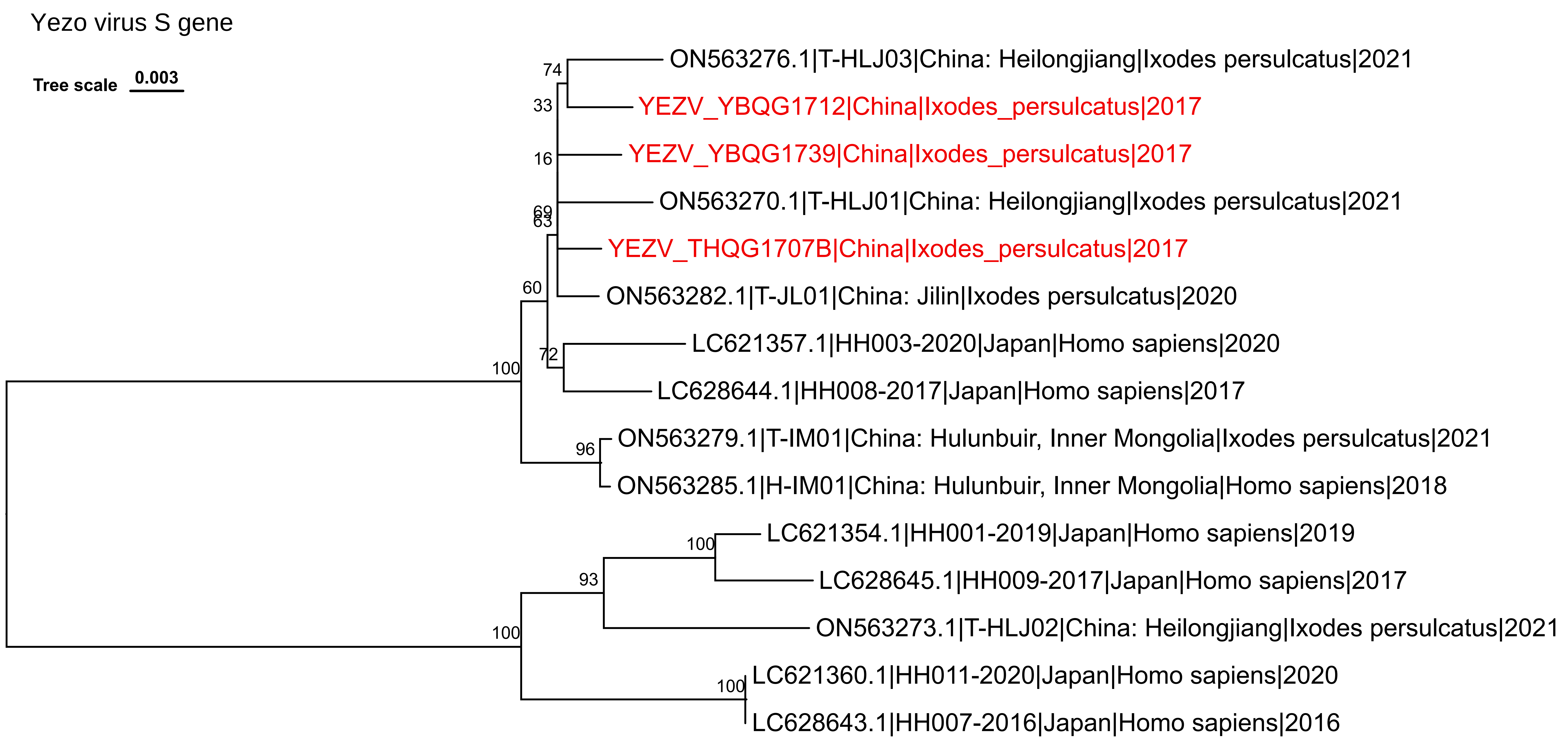
Yezo virus S gene
Tree scale 0.003
ON563276.1|T-HLJ03|China: Heilongjiang|Ixodes persulcatus|2021
YEZV_YBQG1712|China|Ixodes_persulcatus|2017
YEZV_YBQG1739|China|Ixodes_persulcatus|2017
ON563270.1|T-HLJ01|China: Heilongjiang|Ixodes persulcatus|2021
YEZV_THQG1707B|China|Ixodes_persulcatus|2017
ON563282.1|T-JL01|China: Jilin|Ixodes persulcatus|2020
LC621357.1|HH003-2020|Japan|Homo sapiens|2020
LC628644.1|HH008-2017|Japan|Homo sapiens|2017
ON563279.1|T-IM01|China: Hulunbuir, Inner Mongolia|Ixodes persulcatus|2021
ON563285.1|H-IM01|China: Hulunbuir, Inner Mongolia|Homo sapiens|2018
LC621354.1|HH001-2019|Japan|Homo sapiens|2019
LC628645.1|HH009-2017|Japan|Homo sapiens|2017
ON563273.1|T-HLJ02|China: Heilongjiang|Ixodes persulcatus|2021
LC621360.1|HH011-2020|Japan|Homo sapiens|2020
LC628643.1|HH007-2016|Japan|Homo sapiens|2016
74
33
16
69
63
60
72
100
96
100
93
100
100

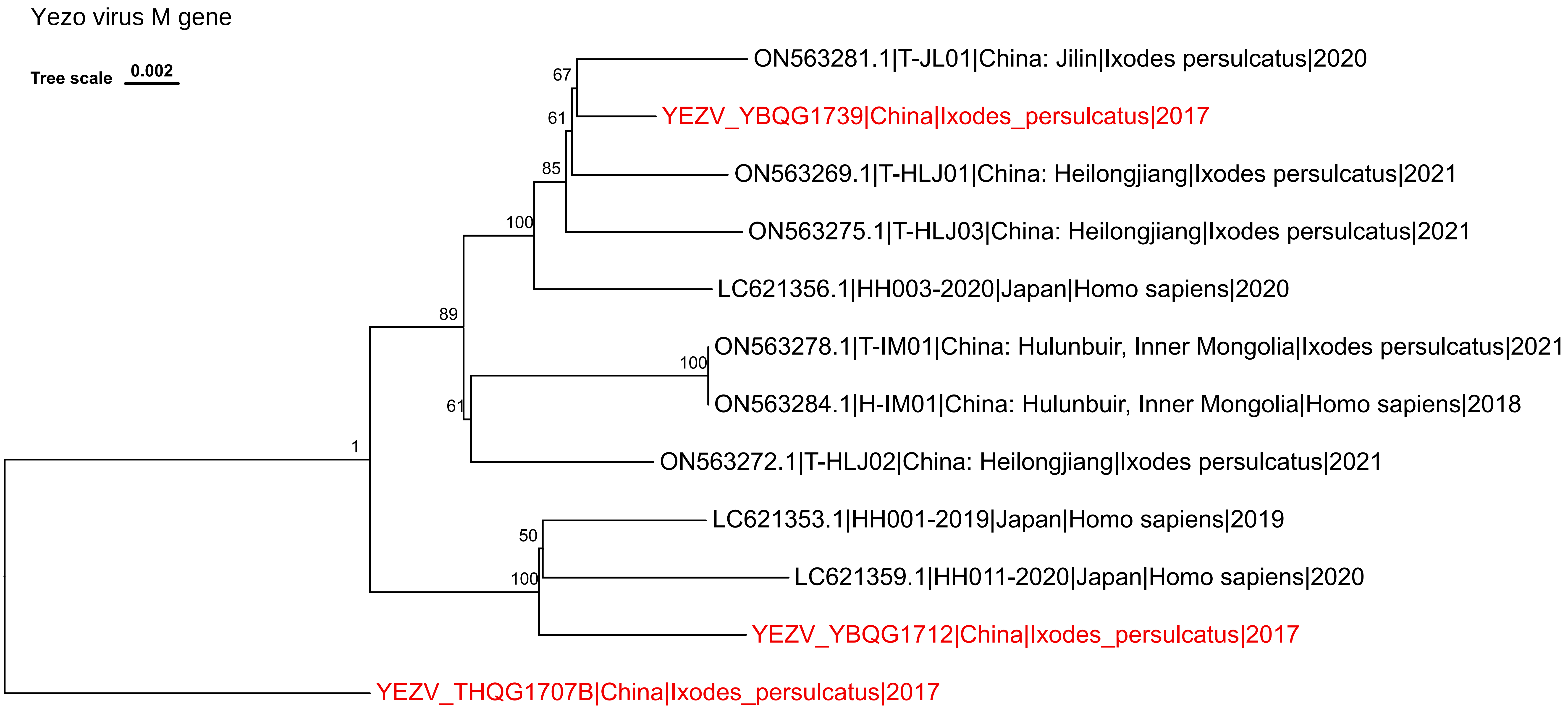

Yezo virus M gene
Tree scale
0.002
ON563281.1|T-JL01|China: Jilin|Ixodes persulcatus|2020
YEZV_YBQG1739|China|Ixodes_persulcatus|2017
ON563269.1|T-HLJ01|China: Heilongjiang|Ixodes persulcatus|2021
ON563275.1|T-HLJ03|China: Heilongjiang|Ixodes persulcatus|2021
LC621356.1|HH003-2020|Japan|Homo sapiens|2020
ON563278.1|T-IM01|China: Hulunbuir, Inner Mongolia|Ixodes persulcatus|2021
ON563284.1|H-IM01|China: Hulunbuir, Inner Mongolia|Homo sapiens|2018
ON563272.1|T-HLJ02|China: Heilongjiang|Ixodes persulcatus|2021
LC621353.1|HH001-2019|Japan|Homo sapiens|2019
LC621359.1|HH011-2020|Japan|Homo sapiens|2020
YEZV_YBQG1712|China|Ixodes_persulcatus|2017
YEZV_THQG1707B|China|Ixodes_persulcatus|2017
67
61
85
100
89
100
61
1
50
100

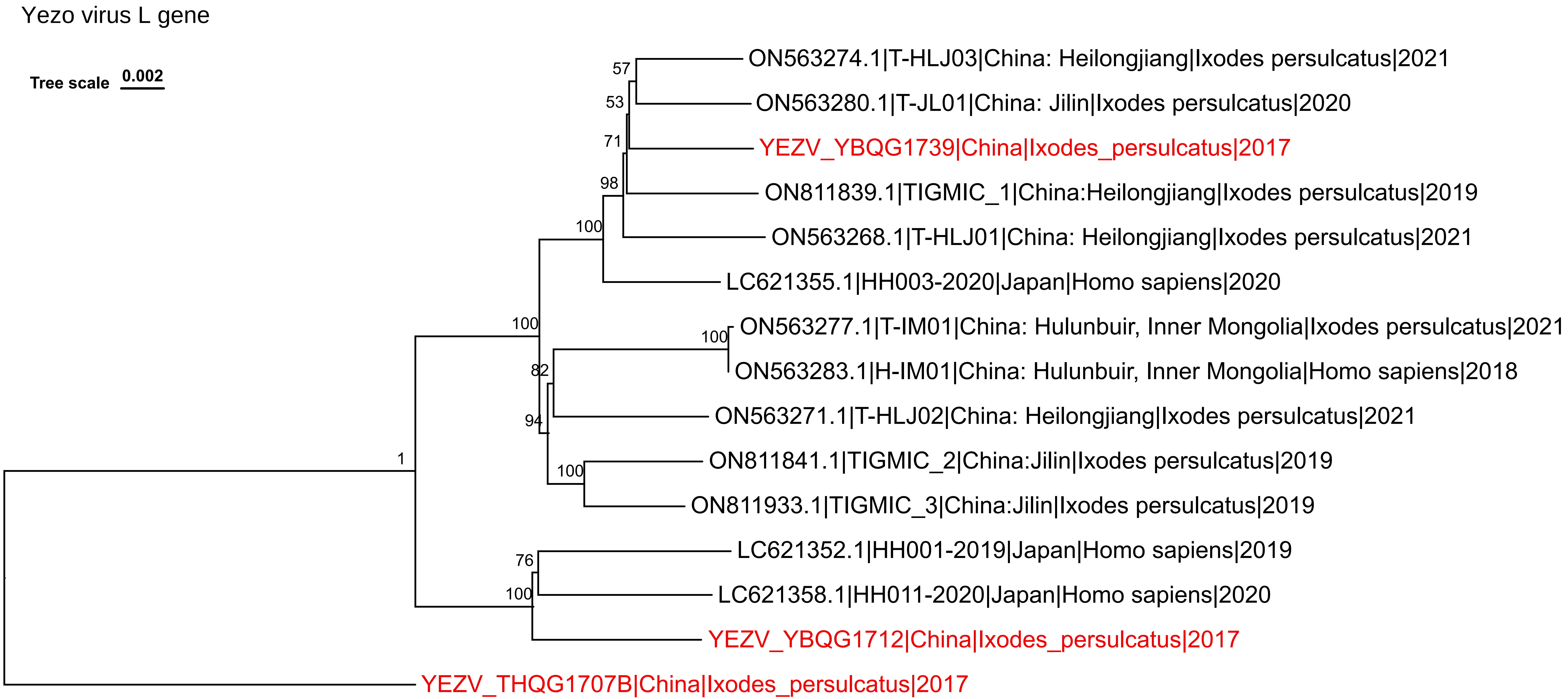

Yezo virus L gene
Tree scale 0.002
ON563274.1|T-HLJ03|China: Heilongjiang|Ixodes persulcatus|2021
ON563280.1|T-JL01|China: Jilin|Ixodes persulcatus|2020
YEZV_YBQG1739|China|Ixodes_persulcatus|2017
ON811839.1|TIGMIC_1|China:Heilongjiang|Ixodes persulcatus|2019
ON563268.1|T-HLJ01|China: Heilongjiang|Ixodes persulcatus|2021
LC621355.1|HH003-2020|Japan|Homo sapiens|2020
ON563277.1|T-IM01|China: Hulunbuir, Inner Mongolia|Ixodes persulcatus|2021
ON563283.1|H-IM01|China: Hulunbuir, Inner Mongolia|Homo sapiens|2018
ON563271.1|T-HLJ02|China: Heilongjiang|Ixodes persulcatus|2021
ON811841.1|TIGMIC_2|China:Jilin|Ixodes persulcatus|2019
ON811933.1|TIGMIC_3|China:Jilin|Ixodes persulcatus|2019
LC621352.1|HH001-2019|Japan|Homo sapiens|2019
LC621358.1|HH011-2020|Japan|Homo sapiens|2020
YEZV_YBQG1712|China|Ixodes_persulcatus|2017
YEZV_THQG1707B|China|Ixodes_persulcatus|2017
57
53
71
98
100
100
100
82
94
100
1
76
100

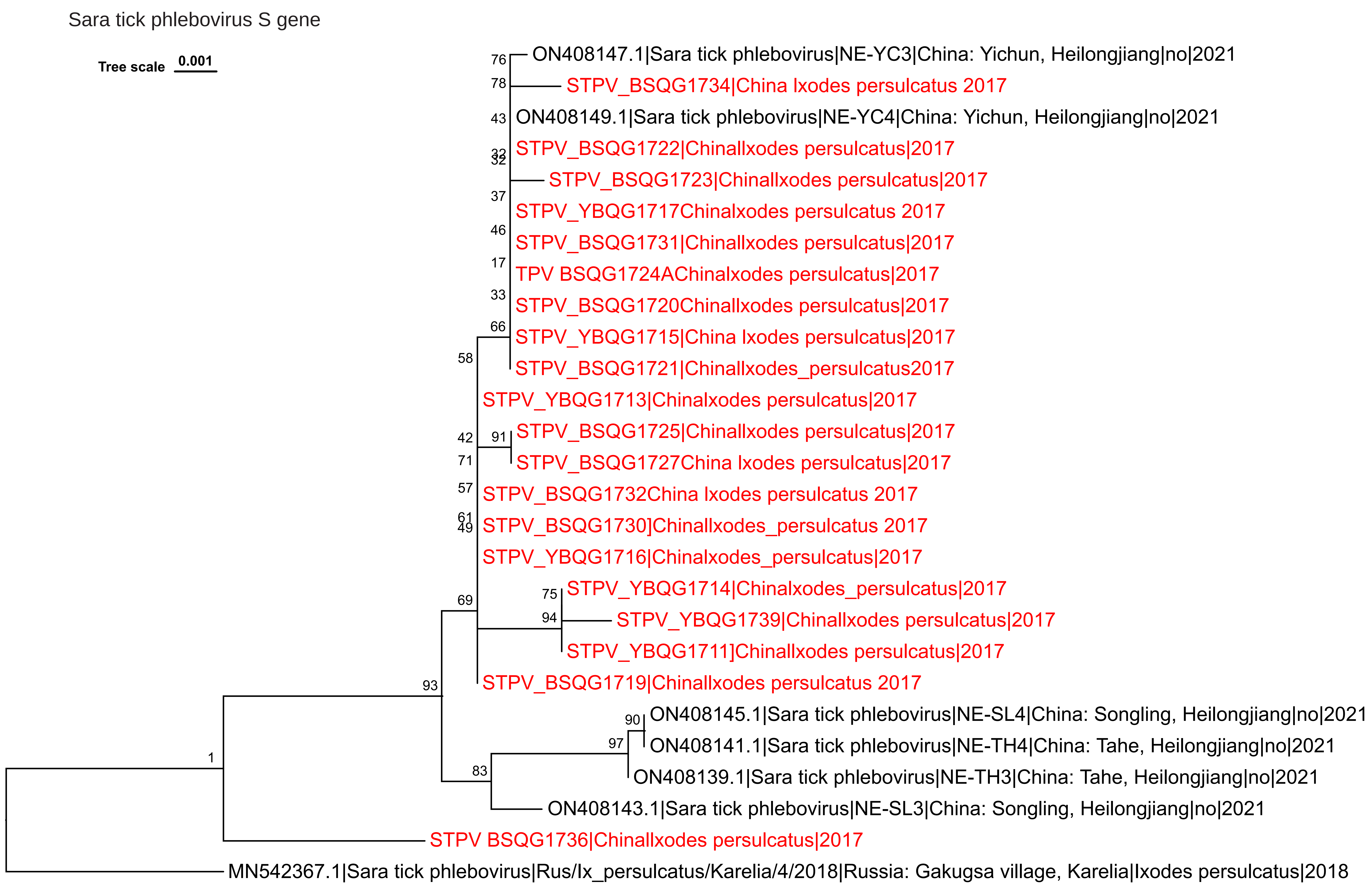

Sara tick phlebovirus S gene
Tree scale 0.001
ON408147.1|Sara tick phlebovirus|NE-YC3|China: Yichun, Heilongjiang|no|2021
STPV_BSQG1734|China Ixodes persulcatus 2017
ON408149.1|Sara tick phlebovirus|NE-YC4|China: Yichun, Heilongjiang|no|2021
STPV_BSQG1722|ChinaIxodes persulcatus|2017
STPV_BSQG1723|ChinaIxodes persulcatus|2017
STPV_YBQG1717ChinaIxodes persulcatus 2017
STPV_BSQG1731|ChinaIxodes persulcatus|2017
TPV BSQG1724AChinaIxodes persulcatus|2017
STPV_BSQG1720ChinaIxodes persulcatus|2017
STPV_YBQG1715|China Ixodes persulcatus|2017
STPV_BSQG1721|ChinaIxodes_persulcatus2017
STPV_YBQG1713|ChinaIxodes persulcatus|2017
STPV_BSQG1725|ChinaIxodes persulcatus|2017
STPV_BSQG1727China Ixodes persulcatus|2017
STPV_BSQG1732China Ixodes persulcatus 2017
STPV_BSQG1730]ChinaIxodes_persulcatus 2017
STPV_YBQG1716|ChinaIxodes_persulcatus|2017
STPV_YBQG1714|ChinaIxodes_persulcatus|2017
STPV_YBQG1739|ChinaIxodes persulcatus|2017
STPV_YBQG1711]ChinaIxodes persulcatus|2017
STPV_BSQG1719|ChinaIxodes persulcatus 2017
ON408145.1|Sara tick phlebovirus|NE-SL4|China: Songling, Heilongjiang|no|2021
ON408141.1|Sara tick phlebovirus|NE-TH4|China: Tahe, Heilongjiang|no|2021
ON408139.1|Sara tick phlebovirus|NE-TH3|China: Tahe, Heilongjiang|no|2021
ON408143.1|Sara tick phlebovirus|NE-SL3|China: Songling, Heilongjiang|no|2021
STPV BSQG1736|ChinaIxodes persulcatus|2017
MN542367.1|Sara tick phlebovirus|Rus/Ix_persulcatus/Karelia/4/2018|Russia: Gakugsa village, Karelia|Ixodes persulcatus|2018

Sara tick phlebovirus L gene

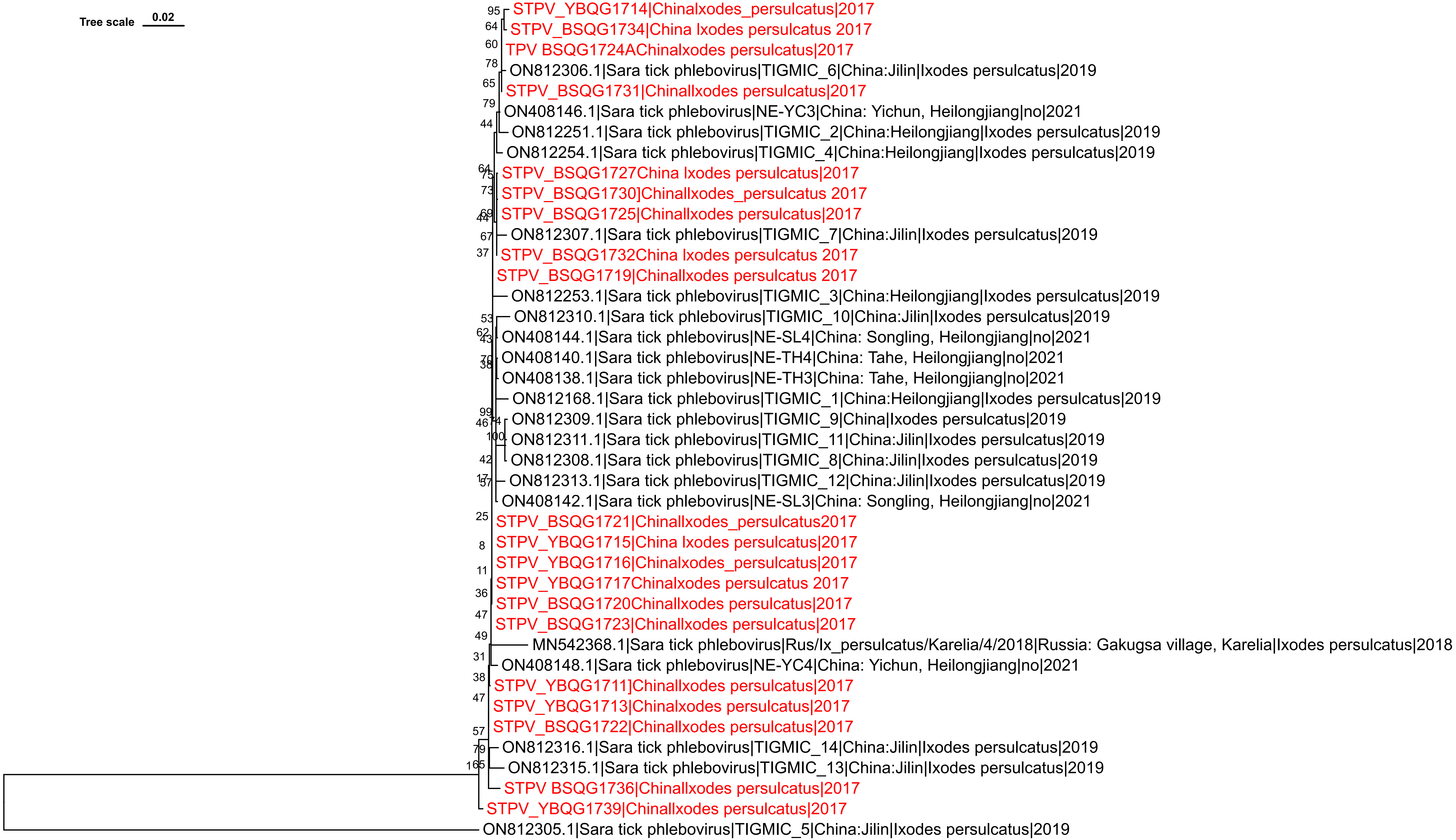

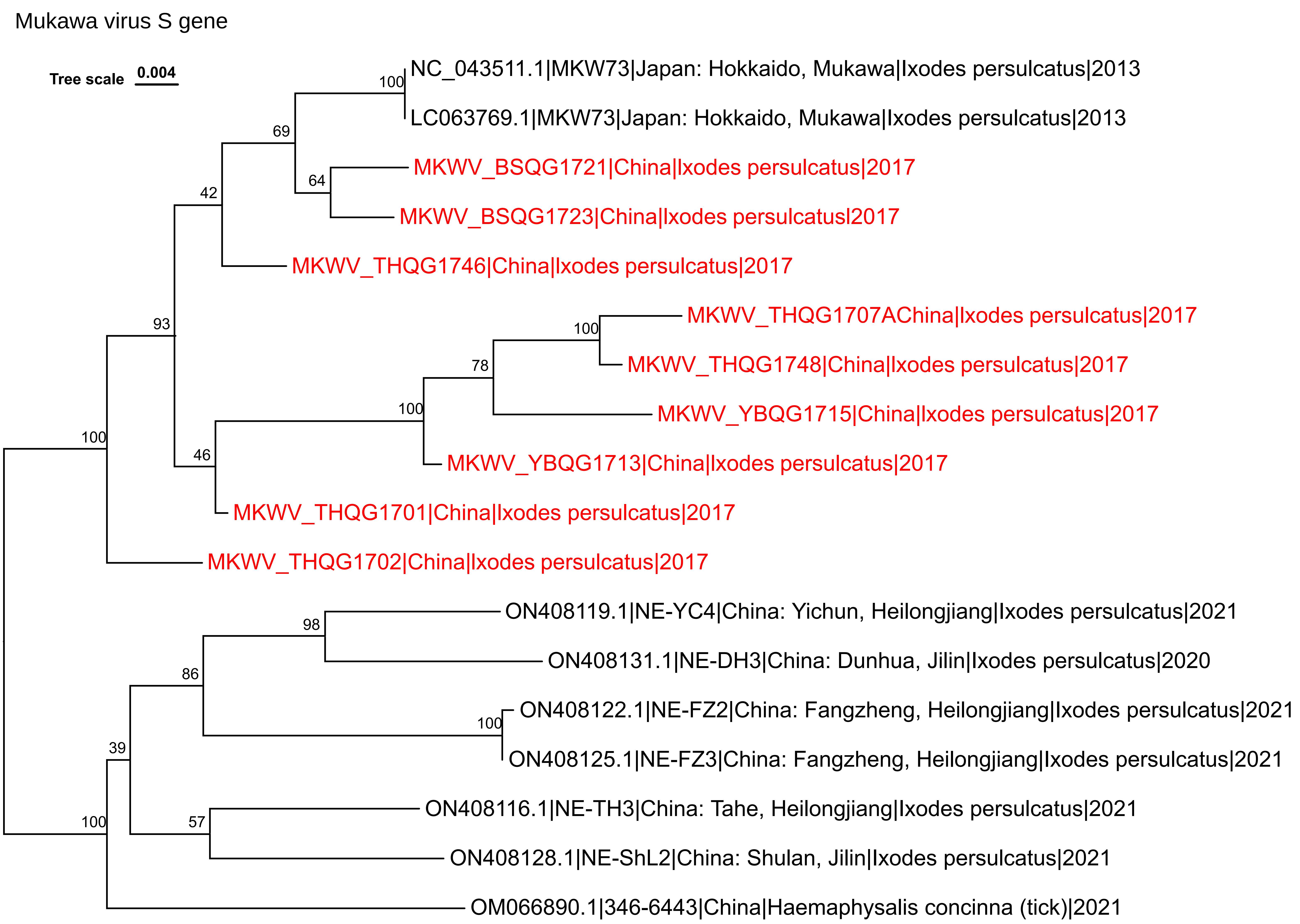
Mukawa virus S gene
Tree scale 0.004
NC_043511.1|MKW73|Japan: Hokkaido, Mukawa|Ixodes persulcatus|2013
LC063769.1|MKW73|Japan: Hokkaido, Mukawa|Ixodes persulcatus|2013
MKWV_BSQG1721|China|Ixodes persulcatus|2017
MKWV_BSQG1723|China|Ixodes persulcatusI2017
MKWV_THQG1746|China|Ixodes persulcatus|2017
MKWV_THQG1707AChina|Ixodes persulcatus|2017
MKWV_THQG1748|China|Ixodes persulcatus|2017
MKWV_YBQG1715|China|Ixodes persulcatus|2017
MKWV_YBQG1713|China|Ixodes persulcatus|2017
MKWV_THQG1701|China|Ixodes persulcatus|2017
MKWV_THQG1702|China|Ixodes persulcatus|2017
ON408119.1|NE-YC4|China: Yichun, Heilongjiang|Ixodes persulcatus|2021
ON408131.1|NE-DH3|China: Dunhua, Jilin|Ixodes persulcatus|2020
ON408122.1|NE-FZ2|China: Fangzheng, Heilongjiang|Ixodes persulcatus|2021
ON408125.1|NE-FZ3|China: Fangzheng, Heilongjiang|Ixodes persulcatus|2021
ON408116.1|NE-TH3|China: Tahe, Heilongjiang|Ixodes persulcatus|2021
ON408128.1|NE-ShL2|China: Shulan, Jilin|Ixodes persulcatus|2021
OM066890.1|346-6443|China|Haemaphysalis concinna (tick)|2021
100
69
64
42
93
100
78
100
46
100
98
86
100
39
57
100

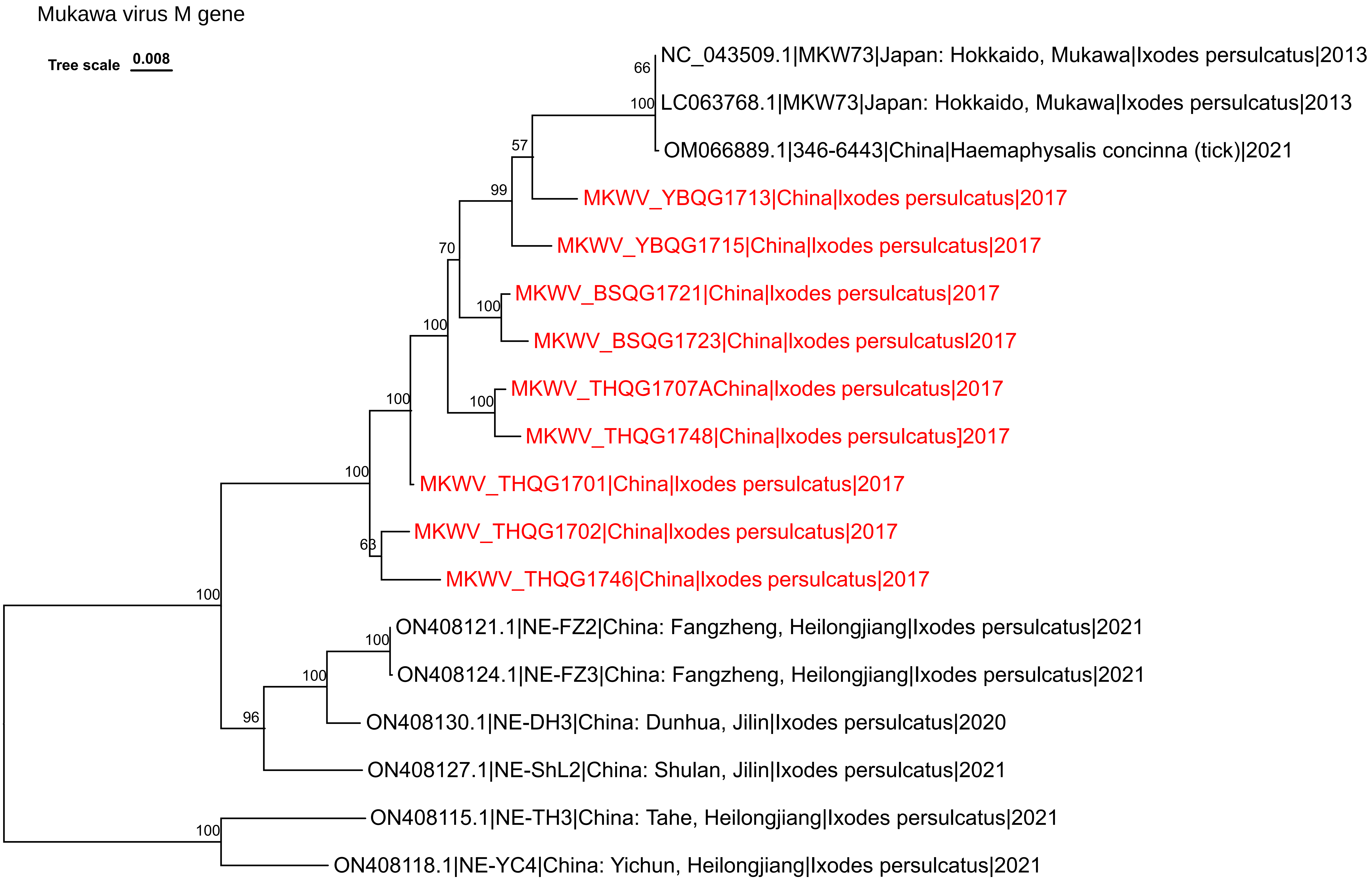
Mukawa virus M gene
Tree scale 0.008
NC_043509.1|MKW73|Japan: Hokkaido, Mukawa|Ixodes persulcatus|2013
LC063768.1|MKW73|Japan: Hokkaido, Mukawa|Ixodes persulcatus|2013
OM066889.1|346-6443|China|Haemaphysalis concinna (tick)|2021
MKWV_YBQG1713|China|Ixodes persulcatus|2017
MKWV_YBQG1715|China|Ixodes persulcatus|2017
MKWV_BSQG1721|China|Ixodes persulcatus|2017
MKWV_BSQG1723|China|Ixodes persulcatusl2017
MKWV_THQG1707AChina|Ixodes persulcatus|2017
MKWV_THQG1748|China|Ixodes persulcatus]2017
MKWV_THQG1701|China|Ixodes persulcatus|2017
MKWV_THQG1702|China|Ixodes persulcatus|2017
MKWV_THQG1746|China|Ixodes persulcatus|2017
ON408121.1|NE-FZ2|China: Fangzheng, Heilongjiang|Ixodes persulcatus|2021
ON408124.1|NE-FZ3|China: Fangzheng, Heilongjiang|Ixodes persulcatus|2021
ON408130.1|NE-DH3|China: Dunhua, Jilin|Ixodes persulcatus|2020
ON408127.1|NE-ShL2|China: Shulan, Jilin|Ixodes persulcatus|2021
ON408115.1|NE-TH3|China: Tahe, Heilongjiang|Ixodes persulcatus|2021
ON408118.1|NE-YC4|China: Yichun, Heilongjiang|Ixodes persulcatus|2021
66
100
57
99
70
100
100
100
100
100
63
100
100
100
96
100

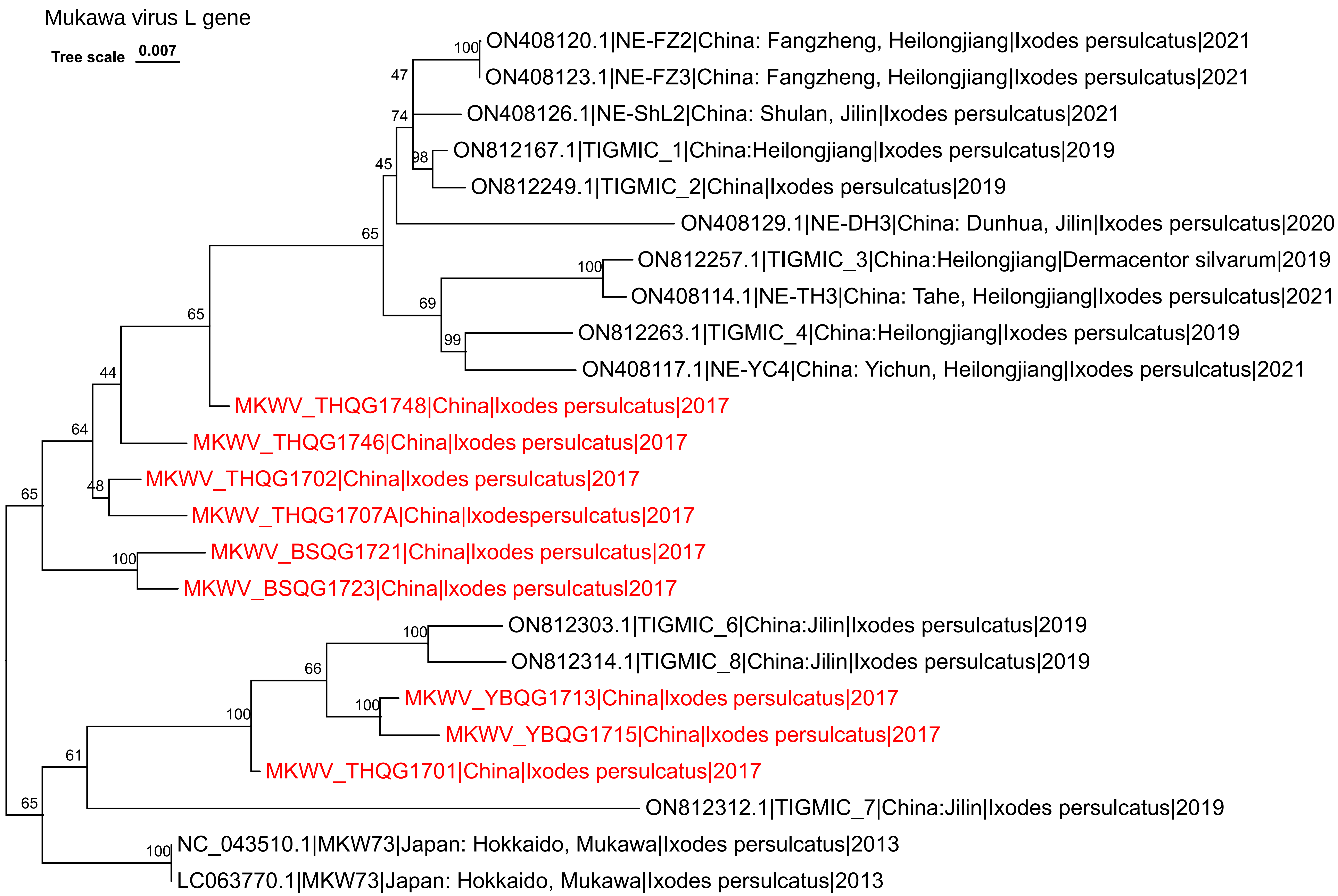
Mukawa virus L gene
Tree scale 0.007
ON408120.1|NE-FZ2|China: Fangzheng, Heilongjiang|Ixodes persulcatus|2021
ON408123.1|NE-FZ3|China: Fangzheng, Heilongjiang|Ixodes persulcatus|2021
ON408126.1|NE-ShL2|China: Shulan, Jilin|Ixodes persulcatus|2021
ON812167.1|TIGMIC_1|China:Heilongjiang|Ixodes persulcatus|2019
ON812249.1|TIGMIC_2|China|Ixodes persulcatus|2019
ON408129.1|NE-DH3|China: Dunhua, Jilin|Ixodes persulcatus|2020
ON812257.1|TIGMIC_3|China:Heilongjiang|Dermacentor silvarum|2019
ON408114.1|NE-TH3|China: Tahe, Heilongjiang|Ixodes persulcatus|2021
ON812263.1|TIGMIC_4|China:Heilongjiang|Ixodes persulcatus|2019
ON408117.1|NE-YC4|China: Yichun, Heilongjiang|Ixodes persulcatus|2021
MKWV_THQG1748|China|Ixodes persulcatus|2017
MKWV_THQG1746|China|Ixodes persulcatus|2017
MKWV_THQG1702|China|Ixodes persulcatus|2017
MKWV_THQG1707A|China|Ixodespersulcatus|2017
MKWV_BSQG1721|China|Ixodes persulcatus|2017
MKWV_BSQG1723|China|Ixodes persulcatusl2017
ON812303.1|TIGMIC_6|China:Jilin|Ixodes persulcatus|2019
ON812314.1|TIGMIC_8|China:Jilin|Ixodes persulcatus|2019
MKWV_YBQG1713|China|Ixodes persulcatus|2017
MKWV_YBQG1715|China|Ixodes persulcatus|2017
MKWV_THQG1701|China|Ixodes persulcatus|2017
ON812312.1|TIGMIC_7|China:Jilin|Ixodes persulcatus|2019
NC_043510.1|MKW73|Japan: Hokkaido, Mukawa|Ixodes persulcatus|2013
LC063770.1|MKW73|Japan: Hokkaido, Mukawa|Ixodes persulcatus|2013
100
47
74
98
45
65
100
69
99
65
44
64
48
65
100
100
66
100
100
61
65
100

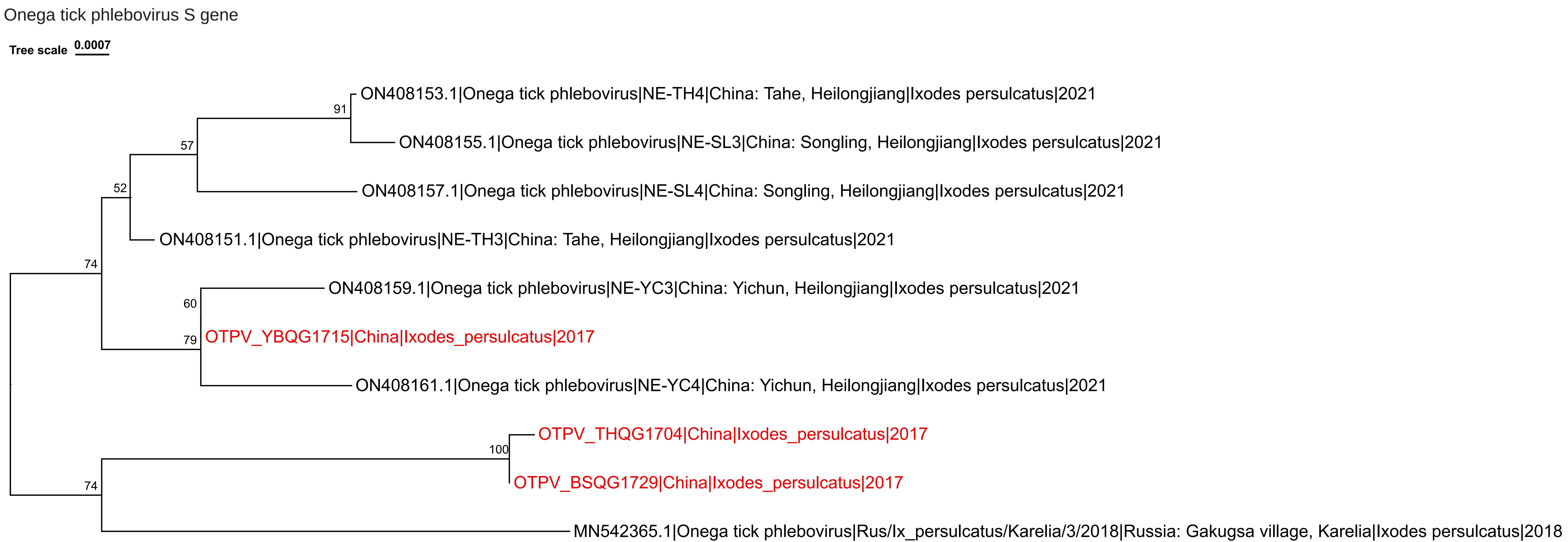
Onega tick phlebovirus S gene
Tree scale 0.0007
ON408153.1|Onega tick phlebovirus|NE-TH4|China: Tahe, Heilongjiang|Ixodes persulcatus|2021
ON408155.1|Onega tick phlebovirus|NE-SL3|China: Songling, Heilongjiang|Ixodes persulcatus|2021
ON408157.1|Onega tick phlebovirus|NE-SL4|China: Songling, Heilongjiang|Ixodes persulcatus|2021
ON408151.1|Onega tick phlebovirus|NE-TH3|China: Tahe, Heilongjiang|Ixodes persulcatus|2021
ON408159.1|Onega tick phlebovirus|NE-YC3|China: Yichun, Heilongjiang|Ixodes persulcatus|2021
OTPV_YBQG1715|China|Ixodes_persulcatus|2017
ON408161.1|Onega tick phlebovirus|NE-YC4|China: Yichun, Heilongjiang|Ixodes persulcatus|2021
OTPV_THQG1704|China|Ixodes_persulcatus|2017
OTPV_BSQG1729|China|Ixodes_persulcatus|2017
MN542365.1|Onega tick phlebovirus|Rus/Ix_persulcatus/Karelia/3/2018|Russia: Gakugsa village, Karelia|Ixodes persulcatus|2018
91
57
52
74
60
79
100
74

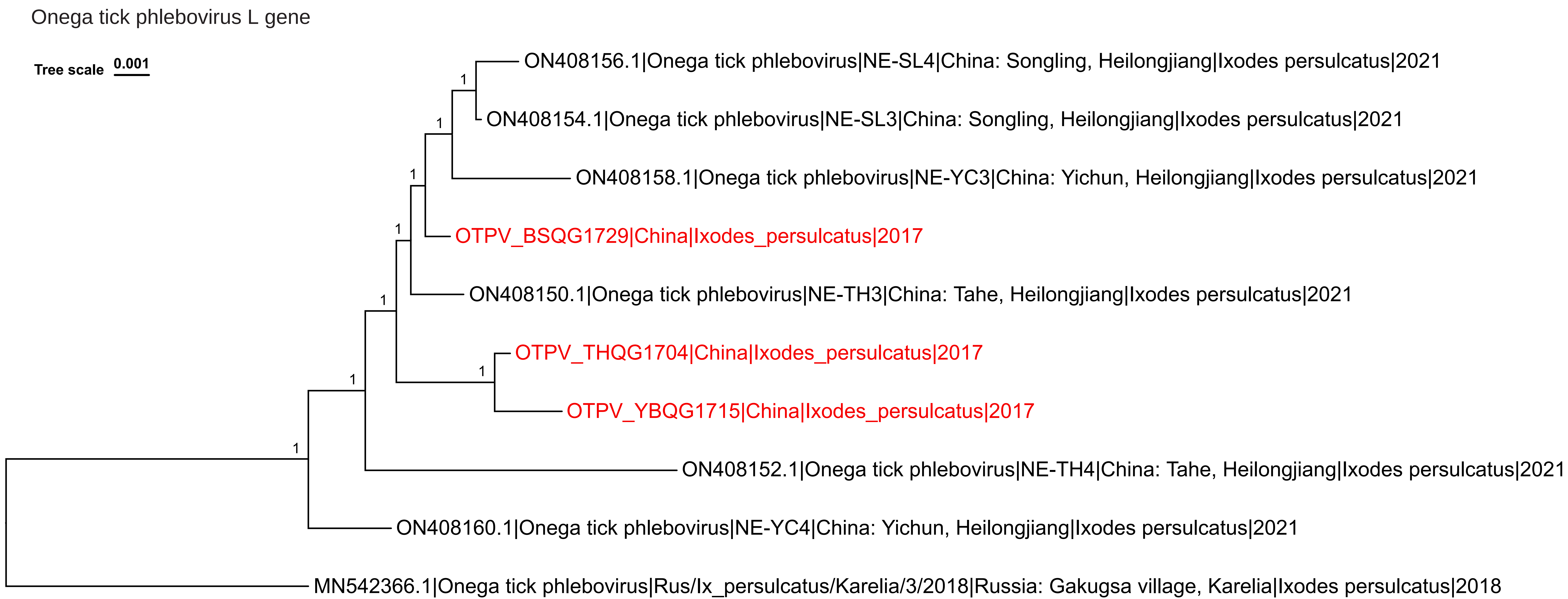
Onega tick phlebovirus L gene
Tree scale
0.001
ON408156.1|Onega tick phlebovirus|NE-SL4|China: Songling, Heilongjiang|Ixodes persulcatus|2021
ON408154.1|Onega tick phlebovirus|NE-SL3|China: Songling, Heilongjiang|Ixodes persulcatus|2021
ON408158.1|Onega tick phlebovirus|NE-YC3|China: Yichun, Heilongjiang|Ixodes persulcatus|2021
OTPV_BSQG1729|China|Ixodes_persulcatus|2017
ON408150.1|Onega tick phlebovirus|NE-TH3|China: Tahe, Heilongjiang|Ixodes persulcatus|2021
OTPV_THQG1704|China|Ixodes_persulcatus|2017
OTPV_YBQG1715|China|Ixodes_persulcatus|2017
ON408152.1|Onega tick phlebovirus|NE-TH4|China: Tahe, Heilongjiang|Ixodes persulcatus|2021
ON408160.1|Onega tick phlebovirus|NE-YC4|China: Yichun, Heilongjiang|Ixodes persulcatus|2021
MN542366.1|Onega tick phlebovirus|Rus/Ix_persulcatus/Karelia/3/2018|Russia: Gakugsa village, Karelia|Ixodes persulcatus|2018
1
1
1
1
1
1
1
1

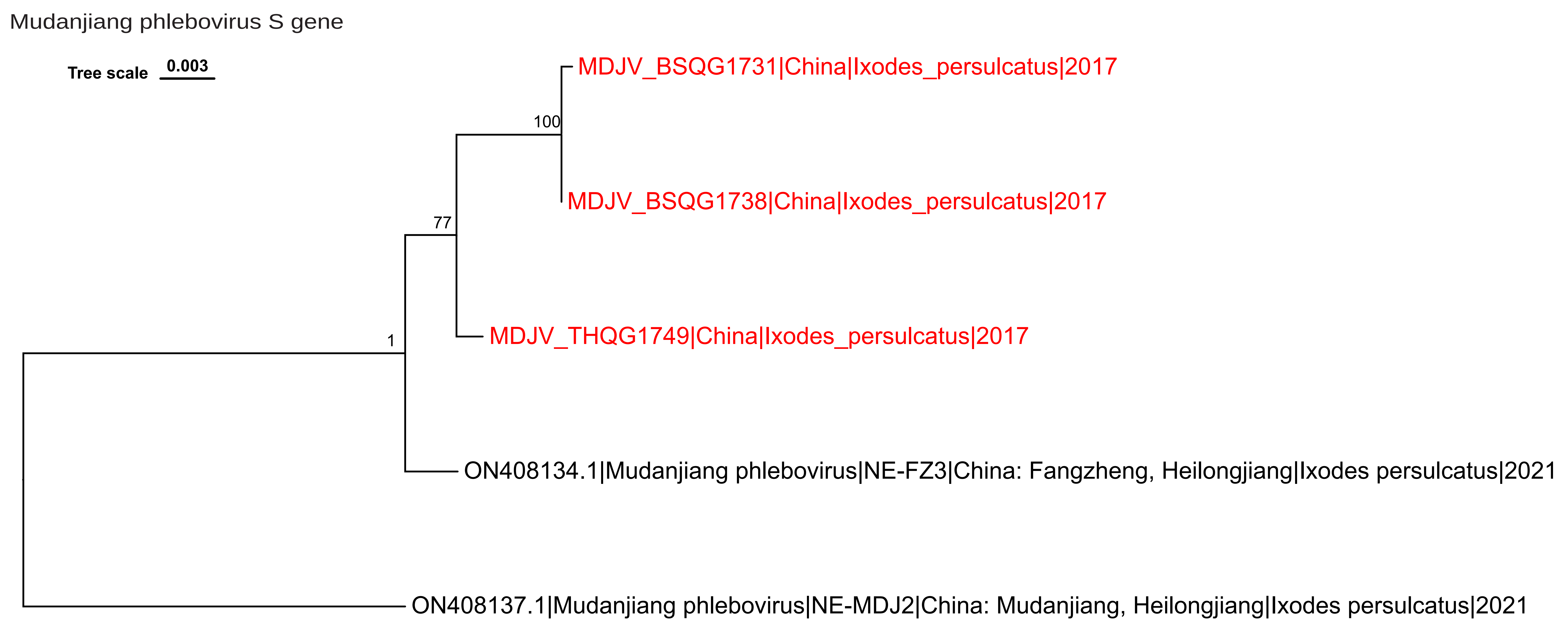

Mudanjiang phlebovirus S gene
Tree scale
0.003
MDJV_BSQG1731|China|Ixodes_persulcatus|2017
100
MDJV_BSQG1738|China|Ixodes_persulcatus|2017
77
MDJV_THQG1749|China|Ixodes_persulcatus|2017
1
ON408134.1|Mudanjiang phlebovirus|NE-FZ3|China: Fangzheng, Heilongjiang|Ixodes persulcatus|2021
ON408137.1|Mudanjiang phlebovirus|NE-MDJ2|China: Mudanjiang, Heilongjiang|Ixodes persulcatus|2021

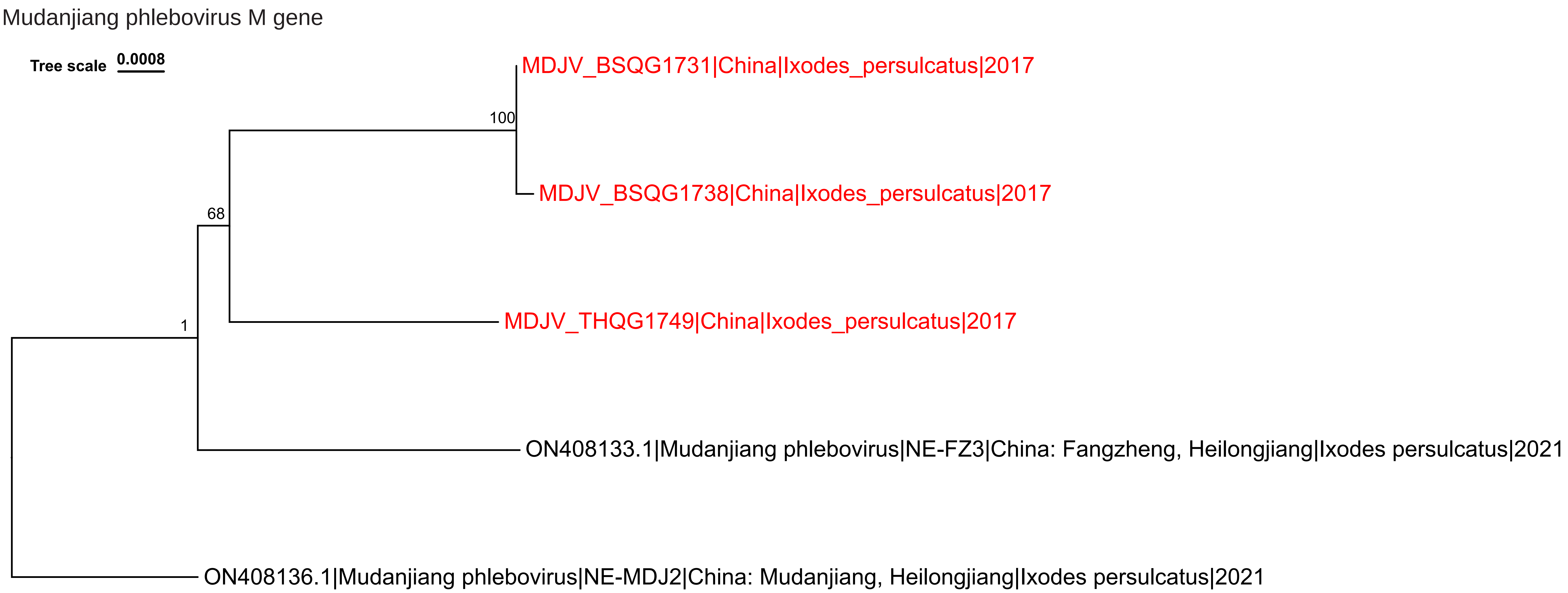
Mudanjiang phlebovirus M gene
Tree scale 0.0008
MDJV_BSQG1731|China|Ixodes_persulcatus|2017
100
MDJV_BSQG1738|China|Ixodes_persulcatus|2017
68
MDJV_THQG1749|China|Ixodes_persulcatus|2017
1
ON408133.1|Mudanjiang phlebovirus|NE-FZ3|China: Fangzheng, Heilongjiang|Ixodes persulcatus|2021
ON408136.1|Mudanjiang phlebovirus|NE-MDJ2|China: Mudanjiang, Heilongjiang|Ixodes persulcatus|2021

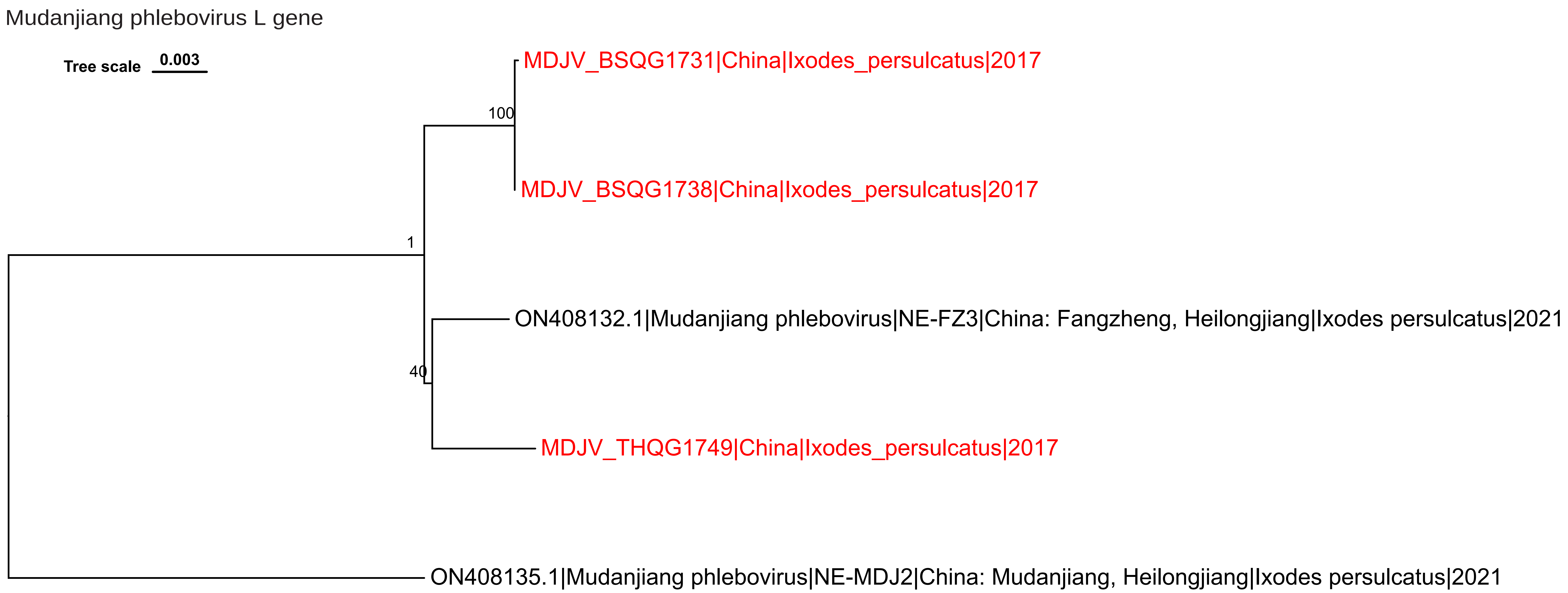
Mudanjiang phlebovirus L gene
Tree scale 0.003
MDJV_BSQG1731|China|Ixodes_persulcatus|2017
100
MDJV_BSQG1738|China|Ixodes_persulcatus|2017
1
ON408132.1|Mudanjiang phlebovirus|NE-FZ3|China: Fangzheng, Heilongjiang|Ixodes persulcatus|2021
40
MDJV_THQG1749|China|Ixodes_persulcatus|2017
ON408135.1|Mudanjiang phlebovirus|NE-MDJ2|China: Mudanjiang, Heilongjiang|Ixodes persulcatus|2021

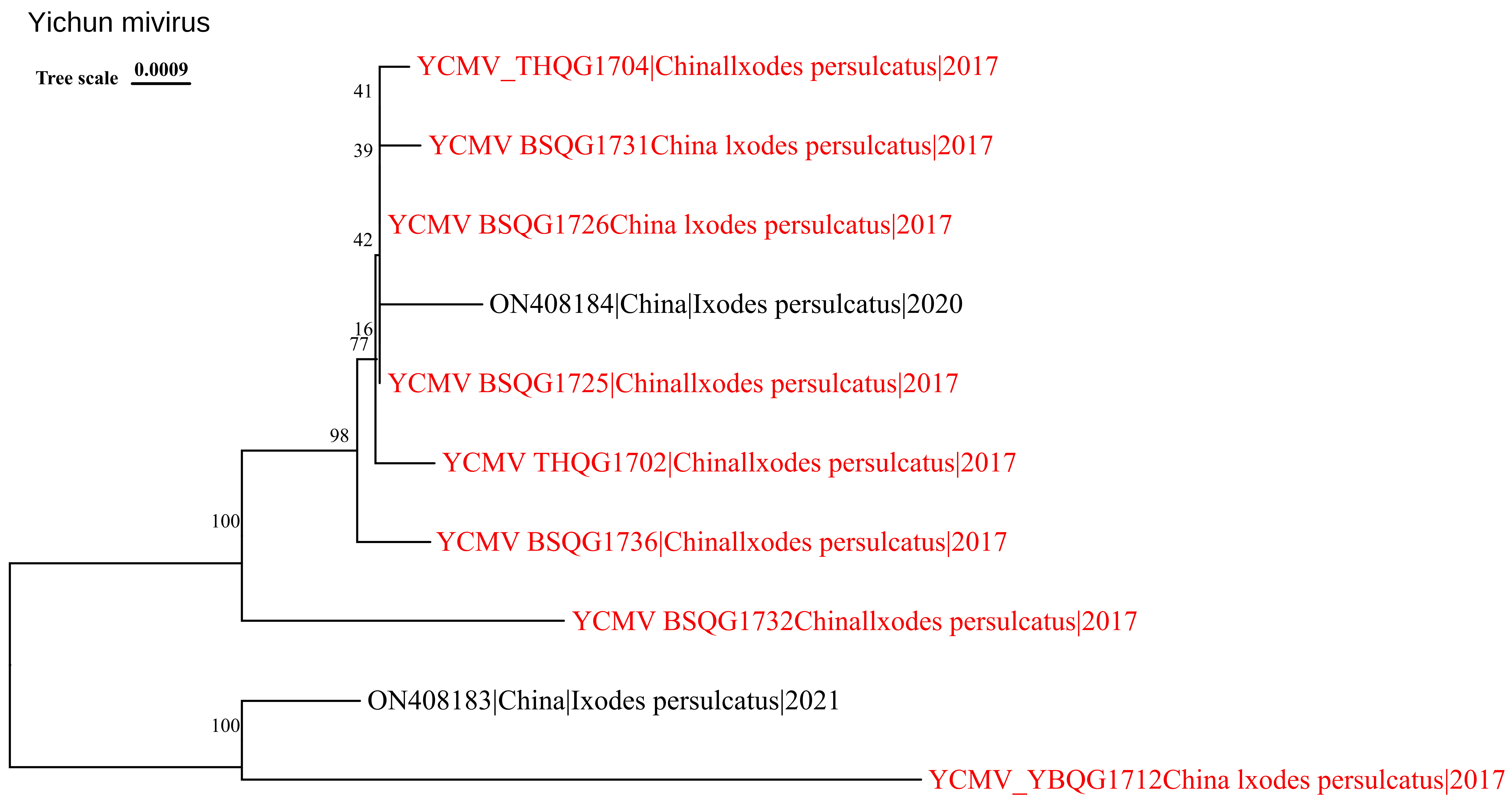
Yichun mivirus
Tree scale 0.0009
YCMV_THQG1704|Chinallxodes persulcatus|2017
YCMV BSQG1731China lxodes persulcatus|2017
YCMV BSQG1726China lxodes persulcatus|2017
ON408184|China|Ixodes persulcatus|2020
YCMV BSQG1725|Chinallxodes persulcatus|2017
YCMV THQG1702|Chinallxodes persulcatus|2017
YCMV BSQG1736|Chinallxodes persulcatus|2017
YCMV BSQG1732Chinallxodes persulcatus|2017
ON408183|China|Ixodes persulcatus|2021
YCMV_YBQG1712China lxodes persulcatus|2017
41
39
42
16
77
98
100
100

Peribunyaviridae sp.

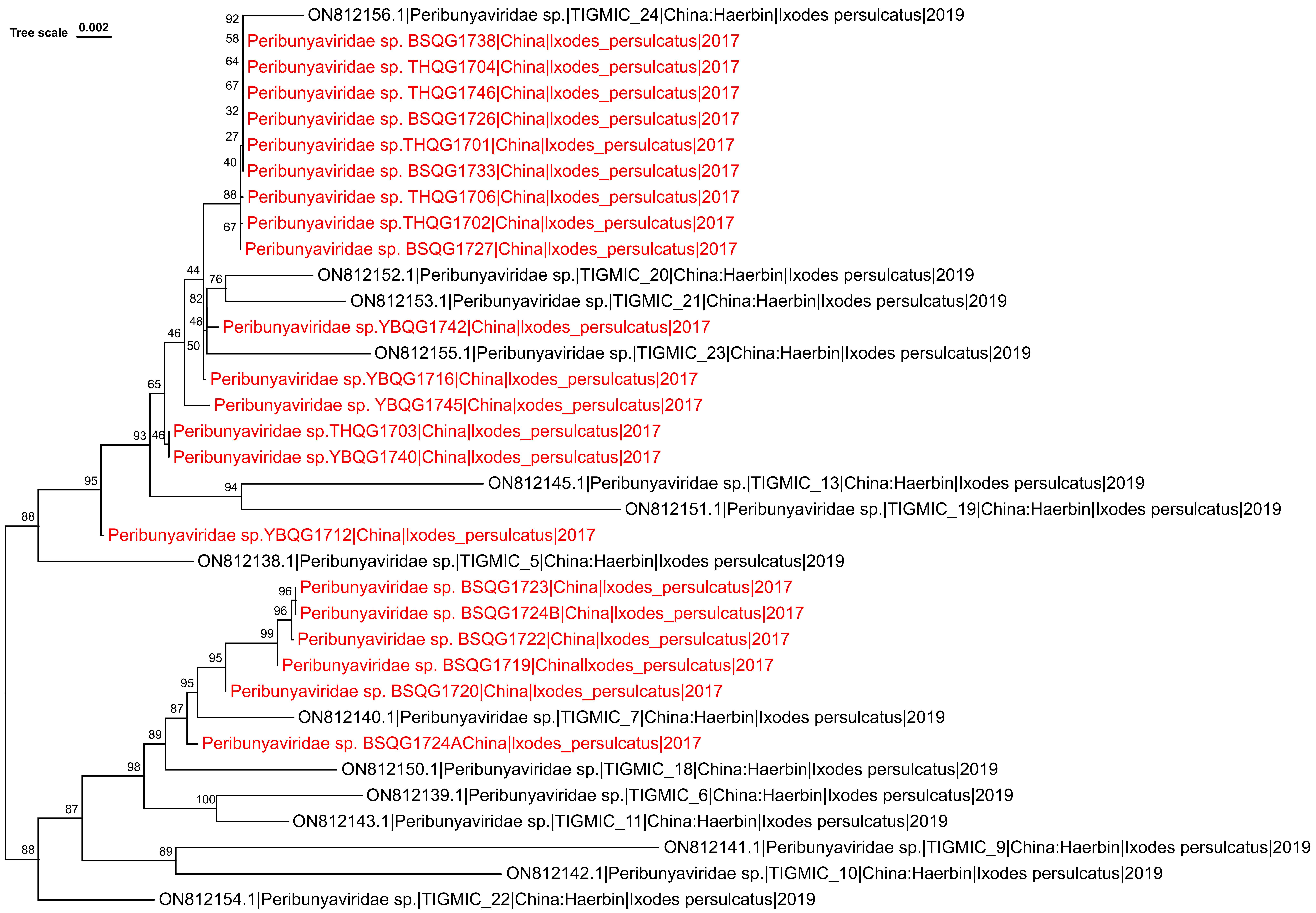

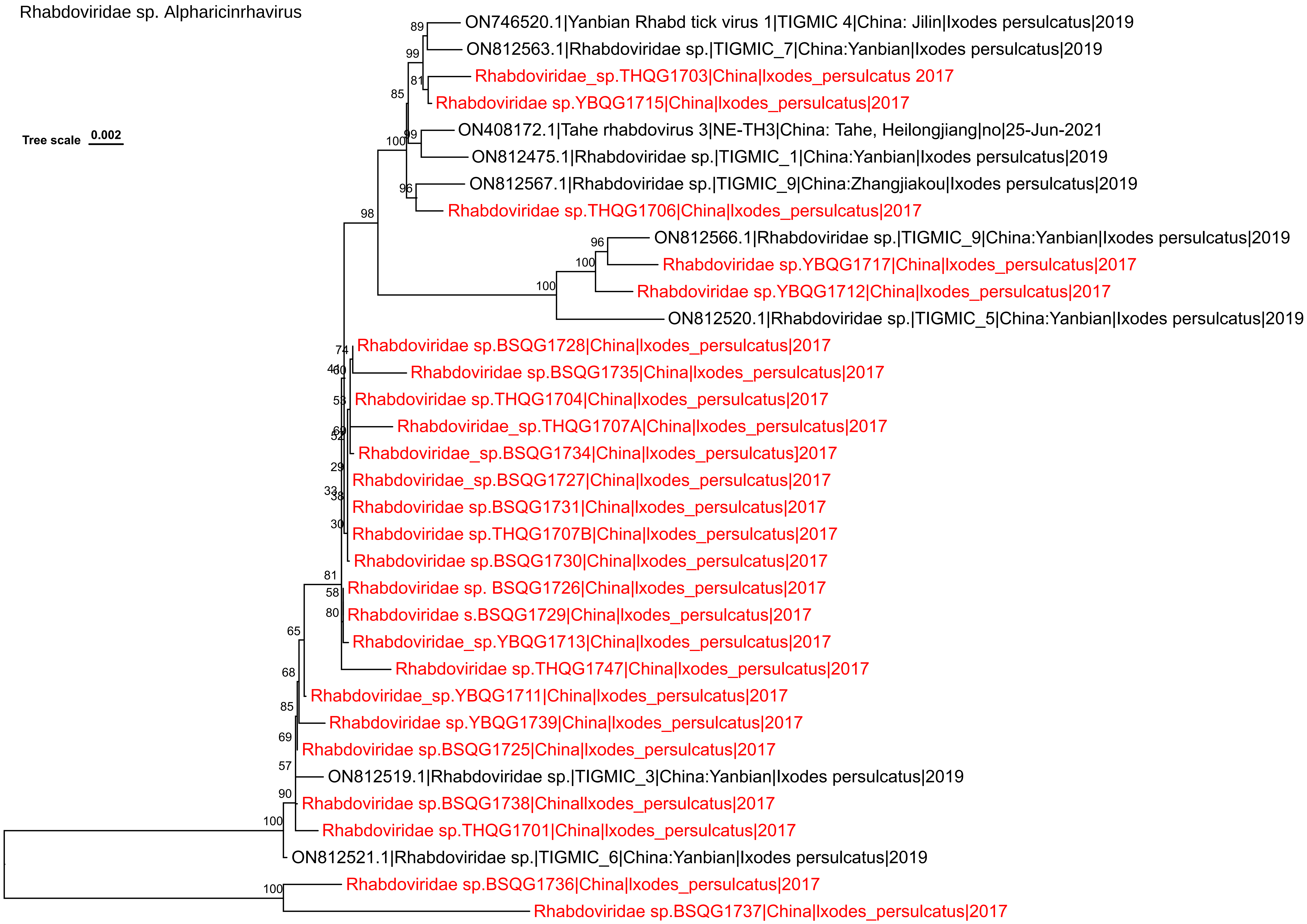
Rhabdoviridae sp. Alpharicinrhavirus
Tree scale 0.002
ON746520.1|Yanbian Rhabd tick virus 1|TIGMIC 4|China: Jilin|Ixodes persulcatus|2019
ON812563.1|Rhabdoviridae sp.|TIGMIC_7|China:Yanbian|Ixodes persulcatus|2019
Rhabdoviridae_sp.THQG1703|China|Ixodes_persulcatus 2017
Rhabdoviridae sp.YBQG1715|China|Ixodes_persulcatus|2017
ON408172.1|Tahe rhabdovirus 3|NE-TH3|China: Tahe, Heilongjiang|no|25-Jun-2021
ON812475.1|Rhabdoviridae sp.|TIGMIC_1|China:Yanbian|Ixodes persulcatus|2019
ON812567.1|Rhabdoviridae sp.|TIGMIC_9|China:Zhangjiakou|Ixodes persulcatus|2019
Rhabdoviridae sp.THQG1706|China|Ixodes_persulcatus|2017
ON812566.1|Rhabdoviridae sp.|TIGMIC_9|China:Yanbian|Ixodes persulcatus|2019
Rhabdoviridae sp.YBQG1717|China|Ixodes_persulcatus|2017
Rhabdoviridae sp.YBQG1712|China|Ixodes_persulcatus|2017
ON812520.1|Rhabdoviridae sp.|TIGMIC_5|China:Yanbian|Ixodes persulcatus|2019
Rhabdoviridae sp.BSQG1728|China|Ixodes_persulcatus|2017
Rhabdoviridae sp.BSQG1735|China|Ixodes_persulcatus|2017
Rhabdoviridae sp.THQG1704|China|Ixodes_persulcatus|2017
Rhabdoviridae_sp.THQG1707A|China|Ixodes_persulcatus|2017
Rhabdoviridae_sp.BSQG1734|China|Ixodes_persulcatus]2017
Rhabdoviridae_sp.BSQG1727|China|Ixodes_persulcatus|2017
Rhabdoviridae sp.BSQG1731|China|Ixodes_persulcatus|2017
Rhabdoviridae sp.THQG1707B|China|Ixodes_persulcatus|2017
Rhabdoviridae sp.BSQG1730|China|Ixodes_persulcatus|2017
Rhabdoviridae sp. BSQG1726|China|Ixodes_persulcatus|2017
Rhabdoviridae s.BSQG1729|China|Ixodes_persulcatus|2017
Rhabdoviridae_sp.YBQG1713|China|Ixodes_persulcatus|2017
Rhabdoviridae sp.THQG1747|China|Ixodes_persulcatus|2017
Rhabdoviridae_sp.YBQG1711|China|Ixodes_persulcatus|2017
Rhabdoviridae sp.YBQG1739|China|Ixodes_persulcatus|2017
Rhabdoviridae sp.BSQG1725|China|Ixodes_persulcatus|2017
ON812519.1|Rhabdoviridae sp.|TIGMIC_3|China:Yanbian|Ixodes persulcatus|2019
Rhabdoviridae sp.BSQG1738|Chinallxodes_persulcatus|2017
Rhabdoviridae sp.THQG1701|China|Ixodes_persulcatus|2017
ON812521.1|Rhabdoviridae sp.|TIGMIC_6|China:Yanbian|Ixodes persulcatus|2019
Rhabdoviridae sp.BSQG1736|China|Ixodes_persulcatus|2017
Rhabdoviridae sp.BSQG1737|China|Ixodes_persulcatus|2017

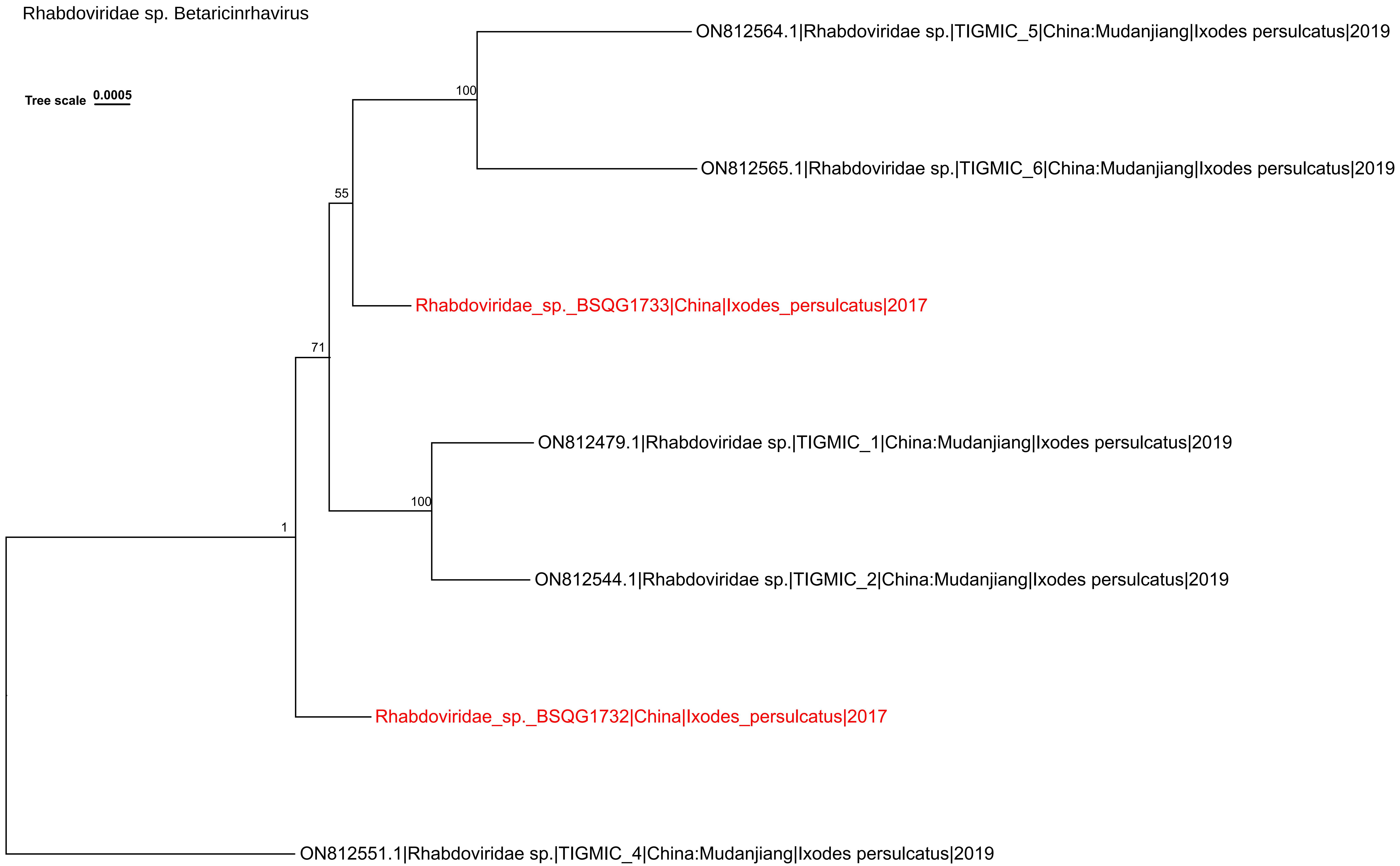
Rhabdoviridae sp. Betaricinrhavirus
Tree scale 0.0005
ON812564.1|Rhabdoviridae sp.|TIGMIC_5|China:Mudanjiang|Ixodes persulcatus|2019
100
ON812565.1|Rhabdoviridae sp.|TIGMIC_6|China:Mudanjiang|Ixodes persulcatus|2019
55
Rhabdoviridae_sp._BSQG1733|China|Ixodes_persulcatus|2017
71
ON812479.1|Rhabdoviridae sp.|TIGMIC_1|China:Mudanjiang|Ixodes persulcatus|2019
100
1
ON812544.1|Rhabdoviridae sp.|TIGMIC_2|China:Mudanjiang|Ixodes persulcatus|2019
Rhabdoviridae_sp._BSQG1732|China|Ixodes_persulcatus|2017
ON812551.1|Rhabdoviridae sp.|TIGMIC_4|China:Mudanjiang|Ixodes persulcatus|2019
