# Supplementary figures and images for: Metatranscriptomics Reveals the RNA Virome of Ixodes Persulcatus in the China–North Korea Border, 2017
Source: Viruses. 2023 Dec 29;16(1):62. doi: 10.3390/v16010062 (PMC10819109; doi:10.3390/v16010062)

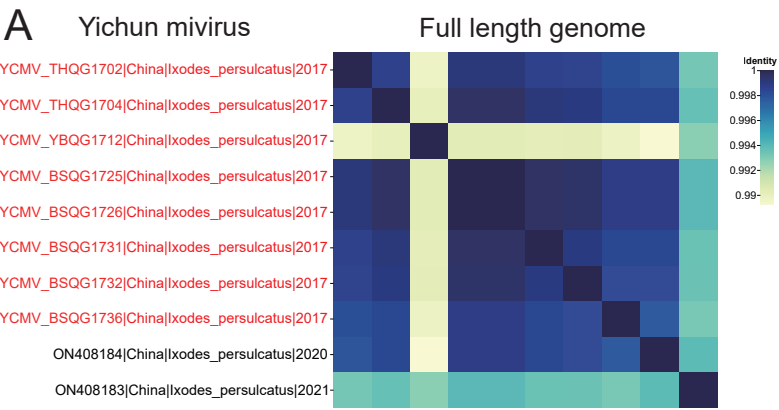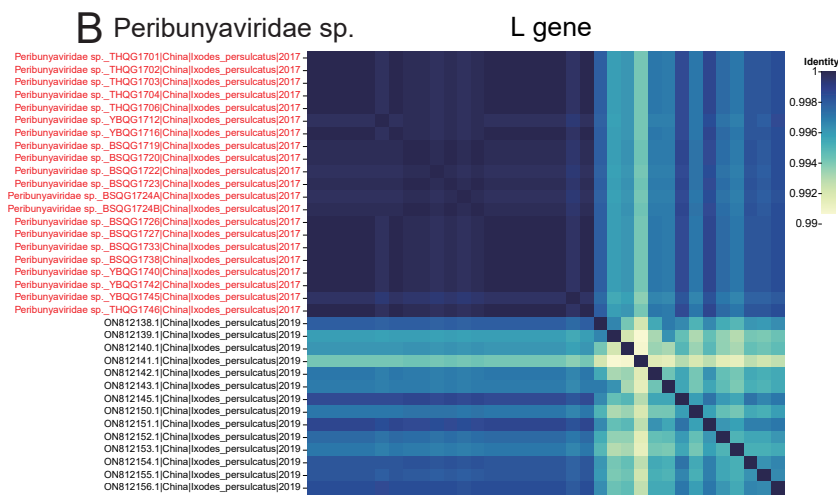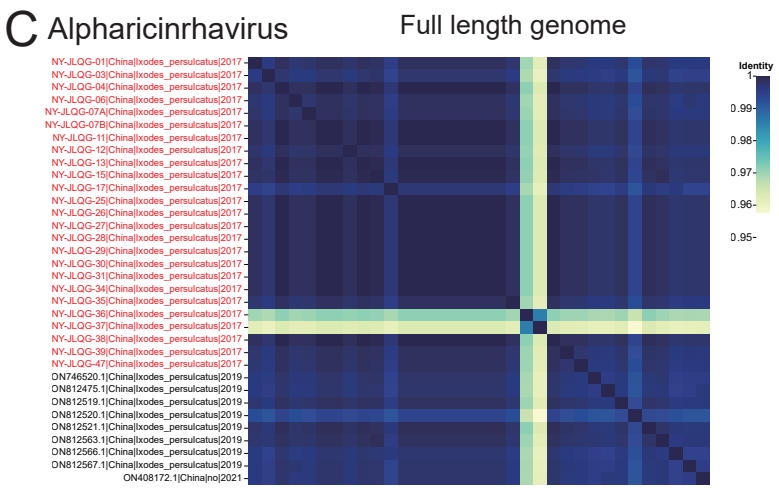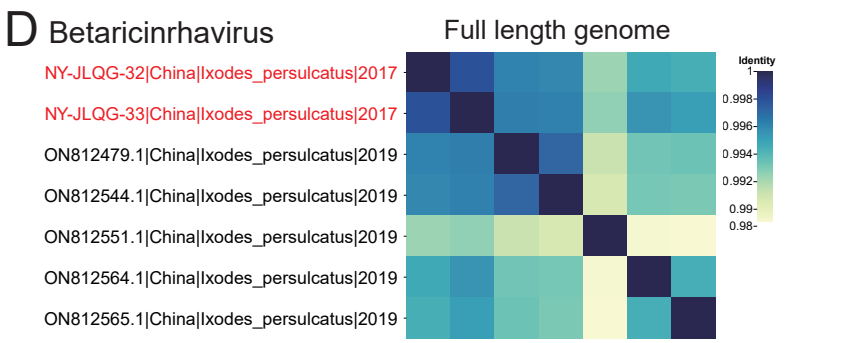

Supplement: Supplementary file 1 [file viruses-16-00062-s001.zip › Figure S2.pdf]
